# B-Modified Pd Cathodes for the Efficient Detoxification of Halogenated Antibiotics: Enhancing C–F Bond Breakage beyond Hydrodefluorination

**DOI:** 10.1021/acs.est.4c12635

**Published:** 2025-03-11

**Authors:** Zefang Chen, Lin Du, Victor Fung, Qingquan Ma, Xiaojun Wang, Shaohua Chen, John C. Crittenden, Yongsheng Chen

**Affiliations:** † School of Civil and Environmental Engineering, Georgia Institute of Technology, Atlanta, Georgia 30332, United States; ‡ CAS Key Laboratory of Urban Pollutant Conversion, Institute of Urban Environment, Chinese Academy of Sciences, Xiamen 361021, P. R. China.; § School of Computational Science and Engineering, Georgia Institute of Technology, Atlanta, Georgia 30332, United States; ∥ Crittenden and Associates, Atlanta, Georgia 30319, United States

**Keywords:** electrocatalytic reduction, Pd-based cathodes, boron doping, C−F bond activation, defluorination, decontamination

## Abstract

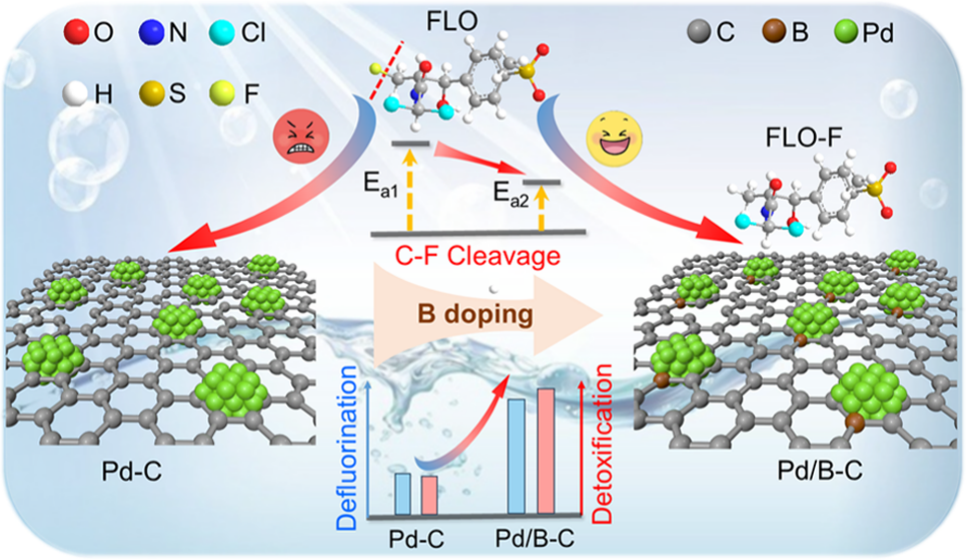

Halogenated antibiotics pose a great threat to aqueous environments because of their persistent biotoxicity from carbon–halogen bonds. Electrochemical reduction (ER) is an efficient technology for dehalogenation, but it still suffers from limited efficiencies in breaking C–F bonds. Herein, we present a strategy to enhance C–F cleavage and promote detoxification by loading benchmark palladium cathodes onto boron-doped carbon. This improves the florfenicol (FLO) degradation rate constant and defluorination efficiency by 1.24 and 1.05 times, respectively, and improves the defluorination of various fluorinated compounds. The cathode with optimal B content shows superior mass activity for FLO degradation (1.11 mmol g^–1^ Pd min^–1^), which is 5.9 times that of commercial Pd/C and is among the best-reported cathodes. Notably, the exclusive formation of the direct defluorination product (i.e., FLO-F) on Pd/B–C implies a higher intrinsic C–F cleavage ability endowed by B doping. As revealed by experiments and theoretical calculations, boron modification enhances palladium binding and induces stronger strain effects and higher electron density for surface palladium atoms, which boosts H* generation and reduces the energy barrier for C–F cleavage. This study provides an effective cathode design strategy to enhance C–F activation, which may broadly benefit the destruction and detoxification of fluorinated organics that are limited by sluggish C–F cleavage kinetics.

## Abstract 2

This study demonstrates boron doping enhances the intrinsic C−F breakage capability of Pd cathodes and explores the underlying mechanisms, with implications for efficient defluorination and detoxification of fluorinated organics

## Introduction

The extensive use of refractory antibiotics, especially halogenated ones, poses a significant threat to human health and the aquatic environment because of their persistent antibacterial activity and biotoxicity from halogen–carbon bonds.^1−6^ Typical oxidation-based methods, although effective for the removal of halogenated antibiotics,^7−9^ may sometimes produce more toxic by-products because of the generation of halogenated by-products or the suboptimal efficiency in cleaving the halogen–carbon bond.^10−17^ Accordingly, there is an urgent need to create efficient and clean approaches for dehalogenation.

Electrochemical reduction (ER) is a cost-efficient and eco-friendly approach that has shown remarkable effectiveness in breaking halogen–carbon bonds and reducing antibacterial activity.^18−20^ Direct electron transfer (DET) and atomic hydrogen (H*) mediated indirect reduction are two major dehalogenation mechanisms in ER.^10,21^ H* is considered more efficient due to a lower required overpotential and higher Faradic efficiency.^10,22^ Palladium (Pd) is an exceptional electrocatalyst for H* generation because of its superior ability to retain H* via adsorption onto the surface and Pd–H bond formation.^2,5,23^ Accordingly, Pd-based electrocatalysts, which possess superior H* generation and storage capability, have been regarded as one of the most effective cathodes for dehalogenation.^4,23−25^ The exceptional dehalogenation efficiency of Pd has also been further advanced through various techniques, including facet engineering,^26^ defect construction,^22^ single-atom formation,^27^ coordination number manipulation,^28^ second metal inclusion,^29,30^ and microenvironment modulation.^31^ However, most reported Pd-based cathodes, although capable of complete dechlorination, have inadequate efficiencies in breaking C–F bonds.^2,5^ This results in considerable remaining antibacterial activity, which is mainly attributed to the unbroken C–F bonds after the electroreduction of fluor-chlorinated antibiotics. Consequently, the deficient efficacy of the C–F bond cleavage is a critical bottleneck to the effective detoxification of halogenated antibiotics.

Breaking the C–F bonds is challenging because of the high bond dissociation energy (BDE, >500 kJ/mol),^32^ necessitating the activation of these bonds by modulating the geometric and electronic structures of Pd-based cathodes. Previous studies have explored various approaches, including microbial systems,^33^ aqueous electron-based photocatalytic reduction systems,^34^ and contact-electrocatalytic systems,^35^ to enhance the C–F bond cleavage. However, these methods often face limitations, such as low efficiency, high energy consumption, and the formation of toxic by-products, which could be potentially addressed by employing a Pd-based ER system. Nevertheless, to the best of our knowledge, no Pd-based cathodes have been specifically designed to boost the C–F breakage. Element doping is a common method to tune the electronic structure of electrocatalysts for a preferred reaction route.^36,37^ The incorporation of boron (B) into Pd-based cathodes, either by subsurface B modification into Pd lattice or by anchoring Pd on a B-doped carbon support, has become an attractive method to improve the performance of Pd-based cathodes.^38−43^ B incorporation can induce the change of both geometric structure (e.g., lattice expansion and strain effect) and electronic structure (e.g., surface core-level shift, d-band center downshift, and charge relocation) for Pd toward enhanced electrocatalytic efficiency and selectivity.^43−47^ Consequently, higher efficiencies in formic acid dehydrogenation,^40,42^ oxygen reduction,^46,47^ and ethanol oxidation,^39^ and enhanced selectivity for CO_2_ electroreduction to formate^43^ and alkyne hydrogenations to *cis*-alkenes^48^ have been observed for Pd-based cathodes with B modification. Additionally, since B has a lower electronegativity than Pd,^43^ B atoms may act as electron donors and thus incur higher electron density for surface Pd atoms.^43,47^ The excess surface electrons may allow more electron transfer to adsorbed fluorinated organics, potentially causing stronger Pd–F interactions and weaker C–F bonds for boosted C–F bond activation and breakage efficiency. Furthermore, the reported B-induced Pd expansion and the resulting enhanced catalytic performance further highlight the potential of B-modified Pd cathodes for improving C–F cleavage.^40,41,43,44,46,48,49^ However, this possibility remains unexplored in the existing literature. This inspires us to explore whether B incorporation into Pd-based cathodes can (i) enhance the C–F breakage performance, (ii) provide a new C–F cleavage mechanism, and (iii) identify the critical electronic and geometric properties induced by B modification that facilitate C–F bond cleavage.

In this work, we aim to verify the hypothesis that B modification on Pd-based cathodes can boost the C–F bond cleavage for higher defluorination efficiency. Pd nanoparticles anchored on both B-doped and undoped carbon supports were fabricated as cathodes to compare their degradation and dehalogenation, especially defluorination, efficiencies on florfenicol (FLO, C_12_H_14_Cl_2_FNO_4_S), a representative toxic halogenated antibiotic that is frequently detected in water matrices and cannot be effectively destroyed by conventional methods.^5,50^ Combining experiments and density functional theory (DFT) calculations, we determined the C–F bond cleavage mechanism and found higher FLO degradation and dehalogenation performance for Pd cathodes with B modification. This study provides an insightful design strategy for electrocatalysts that are targeted at facilitating the C–F bond breakage toward the efficient defluorination of halogenated antibiotics and other fluorinated organics.

## Materials and Methods

### Chemicals and Materials

Detailed information for the materials and chemicals that were used in this study is provided in Text S1.

### Synthesis of Catalysts

B-doped carbon supported palladium catalyst (Pd/B–C, also referred to as Pd/B(0.88)-C when evaluating the role of B) with 4.35 wt % Pd and 0.88 wt % B doping (as determined by inductively coupled plasma optical emission spectroscopy (ICP-OES)) was prepared using the methods that are optimized based on several previous reports.^31,39,51^ First, 1.0 g of carbon black and 5.0 g of H_3_BO_3_ were dispersed in 20 mL of ethanol. The mixture was stirred to dry at 40 °C, ground for 30 min to obtain a uniform mixture, and then heated for 2 h at 900 °C under an H_2_–N_2_ atmosphere to form B–C.

Afterward, 500 mg of the as-obtained B–C was dispersed into a premixed solution, which contained 200 mL of methanol and 115 mg of palladium acetate. The resulting aqueous mixture was then stirred by mixing for 20 min, centrifugally cleaned 3 times with methanol, and vacuum-dried overnight at 60 °C to form Pd/B–C. The homemade Pd/C (ca. 4.49 wt %), which was prepared by the same method without H_3_BO_3_, and commercially available Pd/C (ca. 10.00 wt %, noted as Pd/C (com)) were used for comparison.

To explore the key role of B, two other amounts of H_3_BO_3_ (2 and 10 g) were used to fabricate B–C, which resulted in Pd cathodes with two different B amounts (i.e., 0.58 and 0.71 wt %), denoted as Pd/B(0.58)-C and Pd/B(0.71)-C, respectively. Since the B content in the fabricated cathode slightly decreases as H_3_BO_3_ amounts increase from 5 to 10 g, a H_3_BO_3_ amount of 5 g is considered the optimum amount to fabricate cathodes with the highest B content.

### Characterizations of Catalysts

The morphology and dispersion of Pd/B–C and Pd/C catalysts were characterized by a JEOL JEM-2100F transmission electron microscopy (TEM). A Thermo Scientific Escalab 250Xi X-ray photoelectron spectrometer (XPS) was used to analyze the surface electronic structures using monochromatic Al Kα radiation (1486.68 eV). A Rigaku X-ray diffractometer (XRD) was used to examine crystalline structures with a scan rate of 2° min^–1^ and a step size of 0.01°. Pd K-edge analysis was performed with Si(311) crystal monochromators at the BL14W1 beamlines using Synchrotron Radiation Facility (SSRF). Before the analysis at the beamline, samples were pressed into thin sheets 1 cm in diameter and sealed using the Kapton tape film. The X-ray absorption fine structure (XAFS) spectra were recorded at room temperature using a four-channel silicon drift detector (SDD) Bruker 5040. Pd K-edge extended XAFS (EXAFS) spectra were recorded in the transmission mode. Negligible changes in the line shape and peak position of Pd K-edge X-ray absorption near-edge spectroscopy (XANES) spectra were observed between two scans taken for a specific sample. The XAFS spectra of these standard samples (Pd foil and PdO) were recorded in the transmission mode. The spectra were processed and analyzed by software codes Athena and Artemis. The details for XAFS data processing and wavelet data parameters are provided in Text S2. The chemical composition of the prepared catalysts was analyzed with the aid of ICP-OES (ULTIMA 2, HORIBA JY, FR). The specific surface area was measured by the Brunauer–Emmett–Teller (BET) method using ASAP 2020M+C. The signal of H* trapped by 5,5′-dimethyl-1-pyrroline-oxide (DMPO) (i.e., DMPO-H signal) was detected by electron spin resonance (ESR) on a Bruker model EMX-10/12 spectrometer. The zeta-potential was measured by ZetaPALS. The hydrophobicity of the catalyst surface was determined by the contact-angle measurement (Kruss DSA100).

### Electrochemical Reduction and Analysis

A CHI 760E electrochemical workstation was employed for all electrochemical control and analysis. The electrocatalytic FLO reduction was carried out in an H-type three-electrode reactor (Figure S1), where the cathode and anode chambers were separated by a Nafion 117 membrane. A carbon cloth (1 cm^2^) with a catalyst layer on top was employed as the working electrode. The catalyst layer was obtained by first completely dispersing 2 mg of Pd/B–C, 2 mg of Pd/C, or a proper amount of Pd/C (com) in a 0.5 mL solution that contained 0.125 mL of ethanol, 0.315 mL of water, and 60 μL of 5 wt % Nafion solution to make the catalyst ink. Afterward, 250 μL of the as-acquired catalyst ink was drop-coated onto a cleaned and carbon cloth electrode and dried in an N_2_ atmosphere at room temperature. This resulted in a Pd loading of 43.5–49 μg cm^–2^ on the carbon cloth electrode. A graphite rod electrode (ϕ 6 × 120 mm^2^) and a Hg/Hg_2_SO_4_ electrode served as the counter electrode and the reference electrode, respectively. Various pH values were obtained using 0.2 M phosphate buffer solutions.

For electrochemical characterizations (i.e., electrochemical impedance spectroscopy (EIS), CV, and Cdl), a glassy carbon (GC, ϕ = 5 mm) electrode with 10 μL of the catalyst inks was employed as the working electrode.

The details for a typical run and the electrochemical characterizations are provided in Text S3 and S4, respectively.

### Analytical Methods

The FLO concentrations were measured using high-performance liquid chromatography (HPLC, SHIMADZU-LC20A), and the mobile phase was methanol/H_2_O (48:52, v/v) with a flow rate of 1.0 mL min^–1^. The concentrations of 4-FP, OFX, PFOA, and 5-fluorouracil were all determined by HPLC. The degradation by-products of FLO were identified by a HPLC-triple quadrupole linear ion trap mass spectrometer (Shimadzu LC–20A Prominence/MDS Sciex 3200 Q TRAP). The mobile phase was methanol/0.1% formic acid (1:1, v/v) at a flow rate of 0.3 mL min^–1^. The concentrations of fluoride ion (F^–^) and chloride ion (Cl^–^) were determined by an ion-chromatograph system (ICS-3000, Dionex), and the mobile phase was 20 mM KOH with a flow rate of 1.0 mL min^–1^. The acute toxicity with luminescent bacteria was identified by a SPARK 10 M multimode microplate reader (TECAN, Switzerland).

### Theoretical Simulations

Conformer search was performed by combining Confab,^52^ crest,^53^ xtb,^54−56^ Gaussian 16,^57^ and Molclus^58^ to find the most stable structure of FLO (Figure S2 and Table S1) of FLO among ∼60,000 possible conformers in aqueous solution. Gaussian 16 was employed to calculate bond dissociation energies (BDEs) and standard reduction potentials (E^0^).^57^ All periodic first-principles calculations were performed by the Vienna ab initio simulation package (VASP).^59,60^ The details for all theoretical simulations are provided in Text S5.

### Antibacterial Activity Measurements

The antimicrobial activity of FLO and its dehalogenation by-products were evaluated according to their acute toxicities on luminescent bacteria *Vibrio fischeri* that was resuscitated in advance.^61^ In a typical test, 20 μL of bacteria-containing solution was added into 180 μL samples (10-fold-diluted), 2% NaCl solution (negative control), or sterile water (blank control). The resulting solutions were equilibrated for 15 min in a 96-well plate at 22 °C. The bioluminescence levels of the samples, negative control, and blank control were measured by a multimode microplate reader (SPARK 10 M, TECAN, Switzerland). The inhibition rates based on the changes in luminescence intensity were calculated according to ISO 11348–3:2007. All tests were conducted in triplicate, and the average values with error bars (standard deviations) were reported.

## Results and Discussion

### Characterization

**Figure 1:**
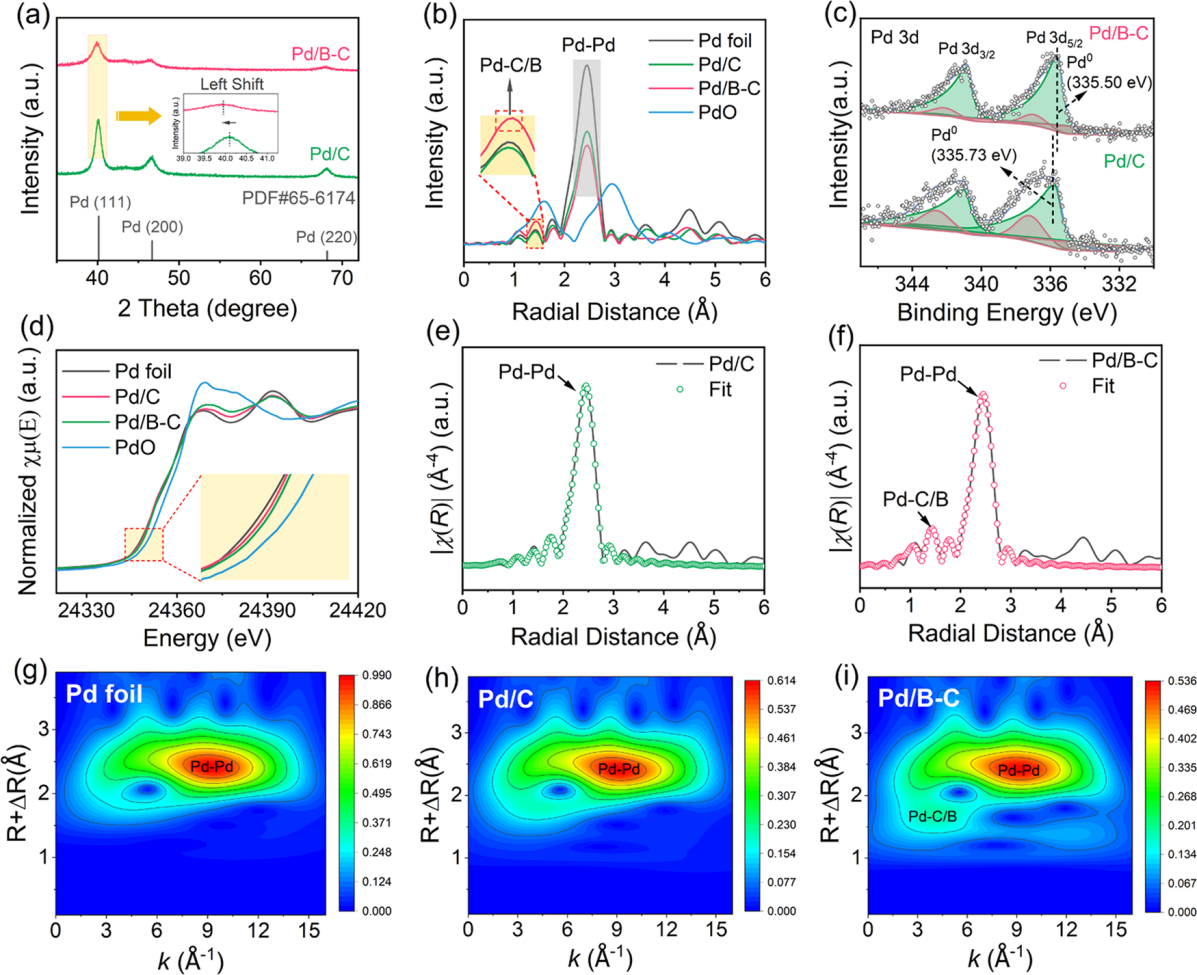
(a) XRD patterns for freshly prepared Pd/B–C and Pd/C. Standard XRD peaks are indicated with vertical bars, according to JCPDS file 65–6174. (b) K-edge EXAFS spectra in R space for Pd/C, Pd/B–C, and references. (c) High-resolution XPS spectra of freshly prepared Pd 3d for Pd/B–C and Pd/C. (d) Pd K-edge normalized XANES spectra of Pd/C, Pd/B–C, and references. EXAFS plots of k^3^.χ phase corrected Fourier transform of experimental and fitted data for (e) Pd/C and (f) Pd/B–C. Pd K-edge wavelet transform contour plots of (g) Pd foil, (h) Pd/C, and (i) Pd/B–C.

The typical TEM images (Figure S3a,e) demonstrate that Pd nanoparticles are well dispersed in both Pd/C and Pd/B–C samples with average diameters of ca. 9.53 ± 1.30 and 7.42 ± 1.26 nm, respectively. The smaller diameter matches well with the higher full width at half-maximum (FWHM) of the Pd(111) diffraction peak for Pd/B–C (Table S2). The more uniform distribution of Pd nanoparticles in Pd/B–C implies the advantage of employing B-doped carbon (B–C) to anchor Pd nanoparticles.^43^ As compared to those for Pd/C, all major XRD diffraction peaks for Pd/B–C shift to smaller angles (Figure 1a), suggesting the expansion of Pd lattice after B doping.^48^ Consistently, Fourier transform EXAFS (FT-EXAFS) analysis (Figures 1e,f and S4) at the Pd K-edge further confirms the higher average Pd–Pd distance for Pd/B–C (2.749 ± 0.011 Å) than that for Pd/C (2.744 ± 0.003 Å) (Table S3). The high-resolution TEM (HRTEM) images (Figure S3c,g) also exhibit a larger average Pd(111) lattice distance for Pd/B–C (0.232 nm) as compared to that for Pd/C (0.228 nm).

As suggested by the best-fit parameters from EXAFS results (Table S3), Pd–O coordination is absent in both Pd/B–C and Pd/C, indicating that Pd mostly presents in nanoparticle forms. Except for this similarity, the fitted EXAFS results of Pd/C appear drastically different from those of Pd/B–C. Pd/C exhibits only one predominant coordination (i.e., the Pd–Pd coordination) with a higher coordination number (i.e., 7.7 ± 0.5) than that for Pd/B–C (6.7 ± 0.4). Notably, Pd/B–C features a distinct coordination between Pd and C or B (thereafter referred as Pd–C/B since the differentiation between Pd–C and Pd–B coordination is unachievable because of the similar bond lengths), which correlates with the exclusive detection of the Pd–C/B peak in the Pd K-edge wavelet transform contour plots of Pd/B–C (Figure 1b,g–i). This implies the stronger interaction between Pd nanoparticles and B–C, which is confirmed by the higher DFT simulated binding energies between Pd and carbon supports with a higher B content (Table S4).

As shown in Figure 1c, the high-resolution XPS peaks of Pd^0^ 3d_5/2_/3d_3/2_ doublets shift from 335.73/340.99 eV to 335.50/340.76 eV after B modification. The red-shift of the Pd^0^ core-level binding energies implies that surface Pd atoms in Pd/B–C would be in a more reduced state compared to those in Pd/C (42), indicating a higher cathodic activity for Pd/B–C. Consistently, DFT calculations suggest that Pd atoms at the surface two layers gain more electrons following B incorporation (Table S5). Nevertheless, the similar peak locations of high-resolution B 1s XPS spectra for B–C and Pd/B–C (Figure S5) indicate that the excess electrons on surface Pd atoms are not likely from B atoms. DFT calculations also find that the change of Bader charge for B atoms is negligible during the process of anchoring Pd on B and C (Table S5). To elucidate the origin of the excess electrons, charge density difference analysis was performed. As demonstrated by Figure S6, B doping in the carbon support causes significantly more electron loss from bulk Pd atoms during the process of anchoring Pd. This is consistent with the higher Pd white line (WL) intensity of Pd/B–C in the XANES spectra (Figure 1d). Meanwhile, a portion of the lost electrons is transferred to surface Pd atoms, and the number of transferred electrons increases with the B content (Table S5). Collectively, B doping in the carbon support induces charge redistribution in Pd nanoparticles during the process of anchoring Pd. This facilitates greater electron transfer from bulk Pd atoms to surface Pd atoms, thereby increasing the electron density of surface Pd atoms. The results of specific surface area and contact-angle measurements suggest that the pore structure and hydrophobicity barely change after B incorporation (Figures S7 and S8).

### Electrocatalytic Performance of Cathodes

**Figure 2:**
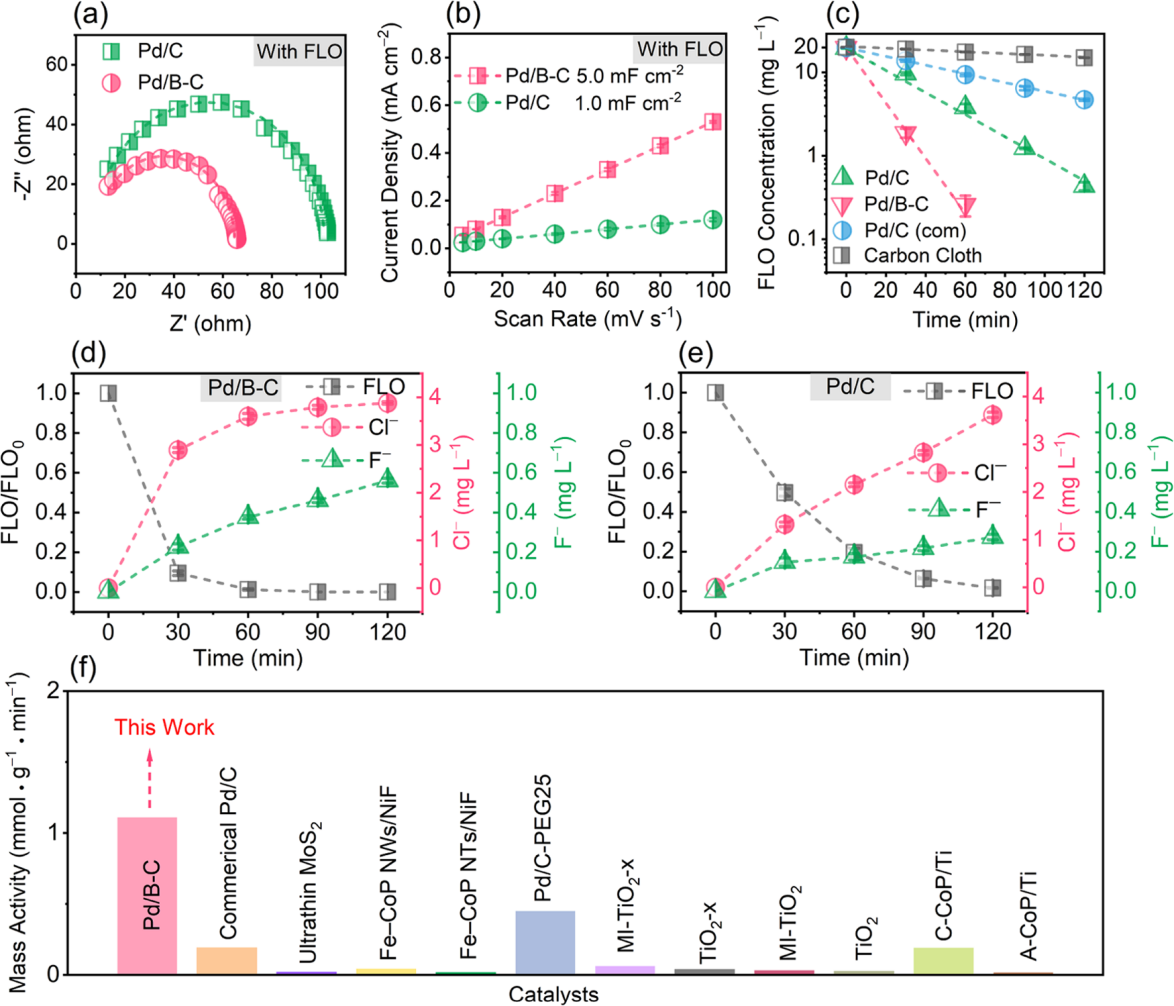
(a) Nyquist plots of Pd/C and Pd/B–C. (b) Linear fitting of the capacitive properties of current density vs scan rate. (c) Concentration profiles of FLO degradation on carbon cloth, Pd/C (com), Pd/C, and Pd/B–C. FLO degradation and released Cl^–^ and F^–^ on (d) Pd/B–C and (e) Pd/C. (f) Comparison of mass activity for electrocatalytic reductive FLO removal (detailed experimental conditions in Table S7). Conditions for (a): 0.1 M Na_2_SO_4_ solution, C_0_ (FLO) = 20 mg L^–1^. Conditions for (c–e): 0.1 M Na_2_SO_4_ solution, C_0_ = 20 mg L^–1^, – 1.0 V vs SHE. For b–e: Data are presented as mean ± s.d. (*n* = 3).

We first evaluated the electrochemical properties for the prepared cathodes in a solution containing 0.1 M Na_2_SO_4_ and 20 mg L^–1^ FLO. As demonstrated by the Nyquist plots (Figure 2a), the charge-transfer resistance (R_ct_) for Pd/B–C (57.4 Ω) is smaller than that of Pd/C (94.6 Ω), indicating a significantly enhanced charge-transfer rate at the electrode/electrolyte interface after B incorporation. The electrochemical double-layer capacitance (*C*_dl_) was acquired by fitting the observed capacitive currents at different scan rates. As shown in Figure 2b, the estimated Cdl for Pd/B–C (5 mF cm^–2^) is 5 times that for Pd/C (1 mF cm^–2^). This suggests a larger electrochemical active surface area (ECSA) and more active sites for Pd/B–C. The electrochemical characterization without FLO is provided in Figures S9–S11 and Table S6.

The electrocatalytic performance of FLO reduction and dehalogenation on different cathodes, including Pd/B–C, Pd/C, and Pd/C (com) with the same Pd loading and bare carbon cloth, was evaluated at −1.0 V vs SHE (i.e., – 1.65 V vs Hg/Hg_2_SO_4_). As shown in Figure 2c, Pd/B–C completely degrades FLO within 90 min, and FLO degradation efficiencies after 60 min of electrolysis and rate constants are of the order of Pd/B–C (98.7%, 4.33 h^–1^) > Pd/C (80.4%, 1.93 h^–1^) > Pd/C (com) (52.6%, 0.73 h^–1^) > carbon cloth (12.5%, 0.15 h^–1^). The estimated mass activity (1.11 mmol g^–1^ Pd min^–1^) and turnover frequency (TOF, 0.57 min^–1^), calculated by eq S5 for Pd/B–C are among the best for cathodic FLO degradation in the literature (Figure 2f and Table S7). Pd/B–C also exhibits the highest dehalogenation efficiency among the four prepared cathodes (Table S8). The released F^–^ (0.56 mg L^–1^, which is 52.8% as compared to complete defluorination) after 120 min of reaction time is 105.1% higher than that for Pd/C (Figure 2d,e). A control experiment without FLO addition excludes the potential contribution of the Nafion membrane or solution to F^–^ production. Subsequently, we evaluated the effects of different factors (i.e., cathode potentials, initial FLO concentrations, and pH values) on FLO dehalogenation using Pd/B–C (details provided in Text S6, Figures S12 and S13, and Table S9). Notably, the exclusive formation of FLO-F (FLO losing one F) for Pd/B–C (Figures S14 and S15) implies a distinct defluorination mechanism, which will be discussed in detail later.

### Mechanism Analysis of the Electrocatalytic Reduction

#### Role of H* and Direct Electron Transfer

Electrocatalytic reductive dehalogenation is mainly achieved by both direct reduction (i.e., direct electron transfer from the cathode to the organics) and indirect reduction (i.e., reduction of organics by H*). To evaluate the contribution of direct electron transfer on FLO reduction, we calculated the standard reduction potential (*E*^0^) for FLO/FLO^–^ (eq S4 in Text S5.3). Since *E*^0^ for FLO/FLO^–^ (−2.03 V vs SHE) is more negative than the potential we applied (i.e., – 1.0 V vs SHE), direct electron transfer may not play an important role in FLO reduction.

**Figure 3:**
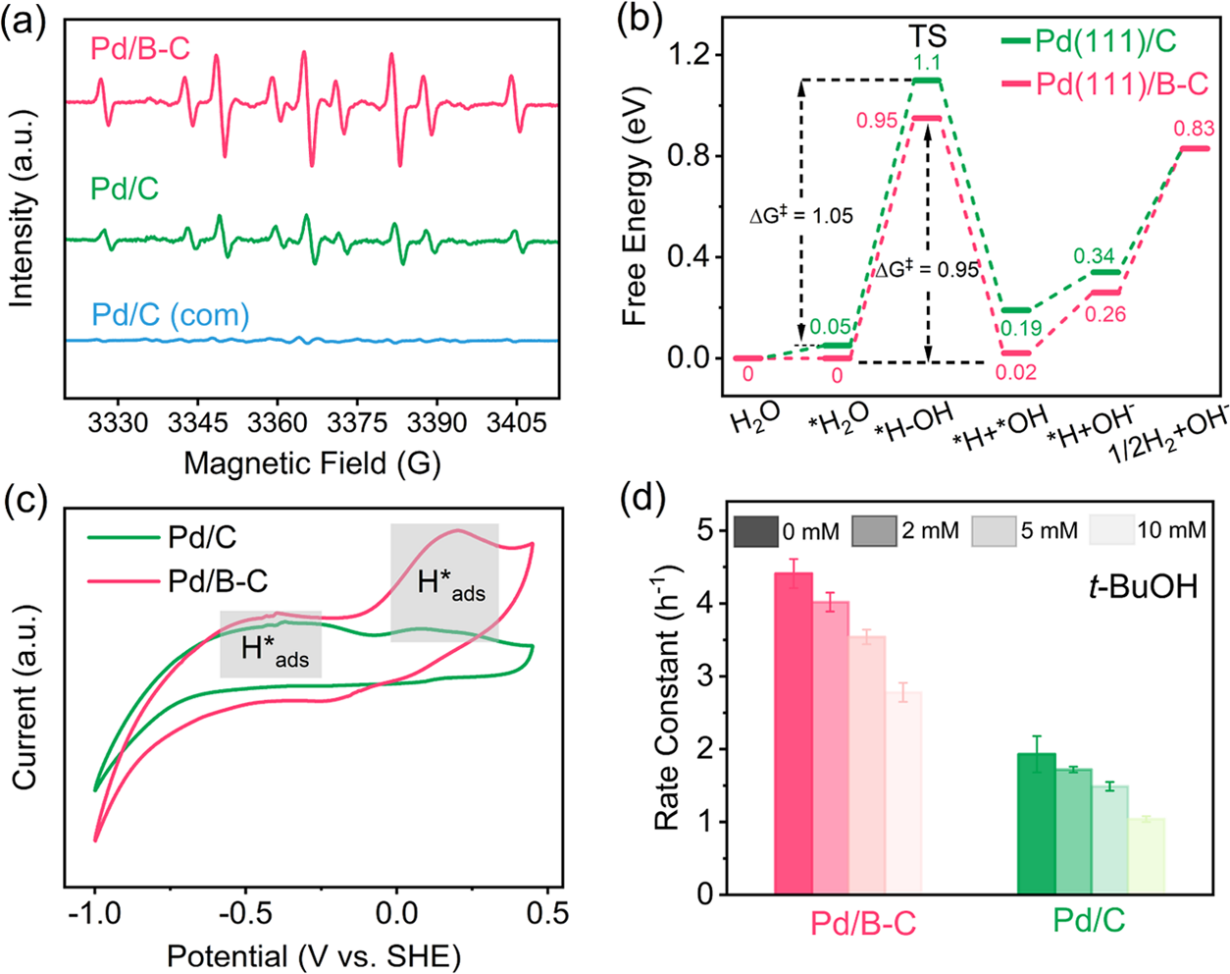
(a) DMPO spin-trapping ESR spectra of Pd/B–C, Pd/C, and Pd/C (com). (b) DFT calculated free energy profiles of H_2_O dissociation on Pd(111)/C and Pd(111)/B–C. (c) CV spectra of Pd/C and Pd/B–C in 0.1 M Na_2_SO_4_ solution (scan rate: 50 mV/s). (d) FLO degradation rate constants on Pd/B–C and Pd/C under different *t*-BuOH concentrations. Conditions for (d): 0.1 M Na_2_SO_4_ solution, *C*_0_ = 20 mg L^–1^, – 1.0 V vs SHE. Data are presented as mean ± s.d. (*n* = 3).

To probe H* generation, ESR measurements were conducted, and the existence of H* is confirmed by the observation of characteristic peaks of DMPO-H adducts (Figure 3a).^31^ The peak intensities are in the order of Pd/B–C > Pd/C > Pd/C (com), suggesting an enhanced H* generation efficiency after B doping. Consistently, DFT simulations reveal that the Pd(111) surface loaded on boron-doped carbon (thereafter referred as Pd(111)/B–C) has a lower Gibbs free energy of activation (Δ*G*^‡^) for H_2_O dissociation (i.e., the reaction that generates H*, 0.95 eV) than the Pd(111) surface loaded on undoped carbon (thereafter referred to as Pd(111)/C, 1.05 eV) (Figure 3b). The optimized configurations of all intermediates involved in H_2_O dissociation on Pd(111)/C and Pd(111)/B–C are presented in Figures S16 and S17. To further differentiate the generation of H species on cathodes, cyclic voltammetry (CV) analysis was performed. As shown in Figure 3c, both Pd/B–C and Pd/C feature the peaks of two H species: (i) absorbed H* (H*_abs_), with the peaks at −0.5 to −0.3 V, and (ii) adsorbed H* (H*_ads_), with the peak at 0.05–0.25 V. By comparing the peak intensities of these two H species, we can conclude that H*_ads_ is the predominant H species for Pd/B–C, and the generation of H*_abs_ and H*_ads_ on Pd/C is comparable. Since only H*_ads_ is responsible for dehalogenation, the much higher H*_ads_ peak for Pd/B–C as compared to that for Pd/C suggests a significantly enhanced H*_ads_ generation efficiency, which correlates with the significantly improved dehalogenation performance after B incorporation.

To further evaluate H* contribution on FLO degradation, *tert*-butyl alcohol (*t*-BuOH) was employed as the scavenger for H*.^62^ As the *t*-BuOH concentration increases from 0 to 10.0 mM, the FLO degradation rate constants for Pd/B–C and Pd/C decrease by 37.0 and 46.1%, respectively (Figure 3d). Additionally, when a cathode with significantly lower Pd loading (10 μL catalyst ink on GC) was used, the FLO degradation efficiency dropped by over 60% at a *t-*BuOH concentration of 10 mM (Figure S18). This highlights the dominant role of *H in FLO degradation. Further evidence is provided by the higher FLO dehalogenation efficiencies observed in solutions with continuous H_2_ feed and the lower efficiencies in O_2_-saturated solutions (Figure S19). Collectively, H* is the primary active species responsible for FLO destruction.

#### DFT Simulations of the C–F Bond Cleavage

The exclusive generation of FLO-F on Pd/B–C suggests a distinct defluorination mechanism that may contribute to better FLO degradation and defluorination performance. To get deep insights into the defluorination mechanisms, DFT calculations were performed to (i) study the electronic structure and surface geometry change after B doping and (ii) simulate the reaction from FLO to FLO-F on Pd(111)/C and Pd(111)/B–C, including no H* and H* preadsorbed conditions. The optimized configurations of intermediates involved in C–F cleavage on Pd(111)/C and Pd(111)/B–C are presented in Figures S20–S23.

**Figure 4:**
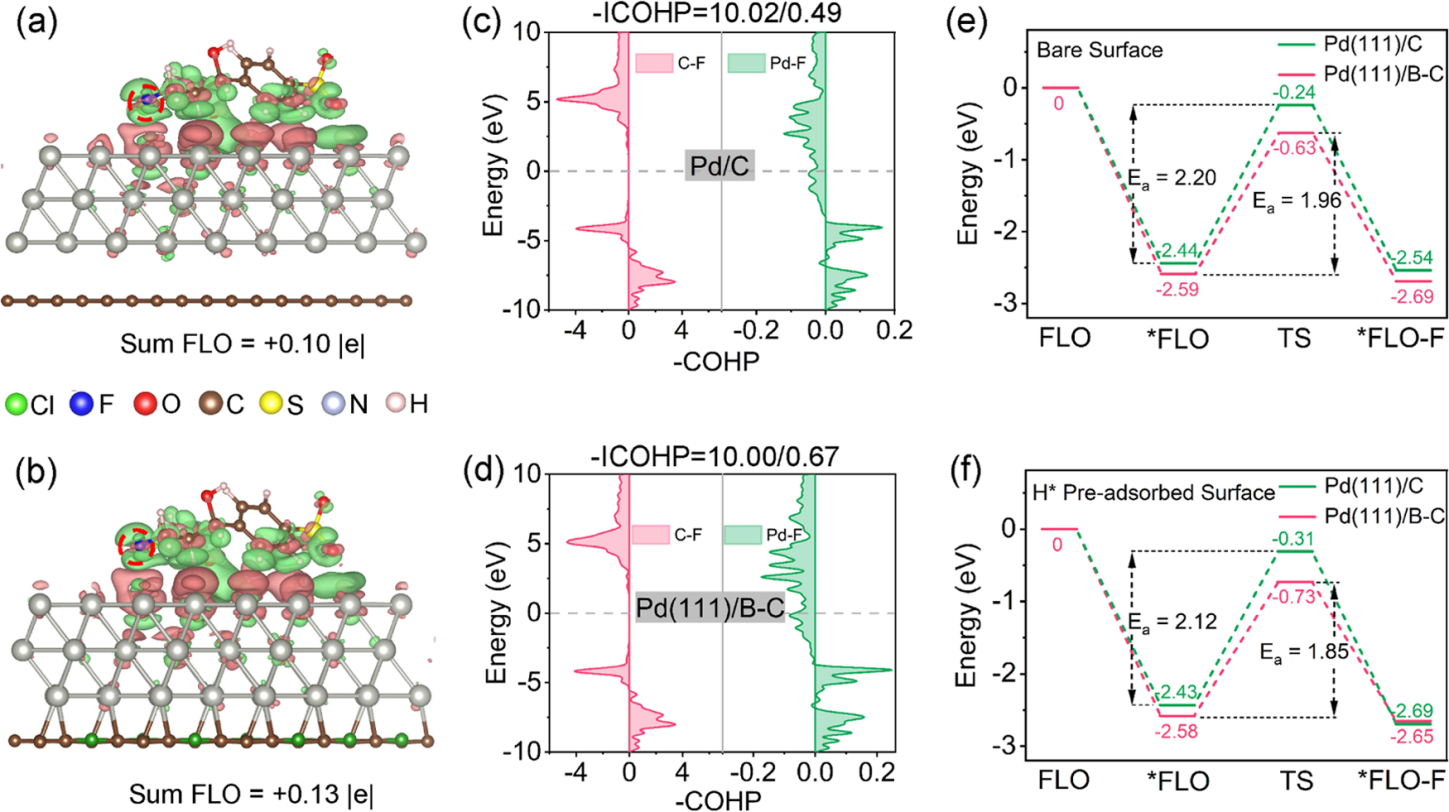
Charge density difference of *FLO on (a) Pd(111)/C and (b) Pd(111)/B–C. Red: charge accumulation. Green: charge depletion. The isosurface value is 0.008 e Å^–3^. COHP of C–F and Pd–F for (c) Pd(111)/C and (d) Pd(111)/B–C. Energy diagram of the adsorbed FLO and defluorination (FLO-F) process on (e) bare surfaces and (f) H* preadsorbed surfaces.

DFT results suggest a larger Pd–Pd distance of surface Pd atoms (i.e., a stronger strain effect) for Pd(111)/B–C than that for Pd(111)/C (Table S10), indicating a possibly higher activation of adsorbed FLO. As shown in Figure 4a,b, more electrons are transferred to FLO molecules than are adsorbed on Pd(111)/B–C (0.130 |e|) as compared to that on Pd(111)/C (0.10 |e|). This correlates with the higher electron density on surface Pd atoms of Pd(111)/B–C and suggests a possibly higher activation of adsorbed FLO molecules. Consequently, the C–F bond length of the adsorbed FLO increases from 1.41 to 1.42 Å, and the distance between the F atom and the closest Pd atom drastically decreases from 3.24 to 3.07 Å. This suggests a slightly weaker C–F bond and a stronger interaction between the Pd and F atoms, potentially leading to a more favorable defluorination reaction. To further support this deduction, crystal orbital Hamiltonian populations (COHP) for C–F and Pd–F were studied to evaluate the interactions between F atoms and both Pd and C atoms (Figure 4c,d). The more negative ICOHP value at the Fermi level for Pd–F suggests strong interactions between Pd and F atoms after B incorporation. A weaker C–F bond is also confirmed from the less negative ICOHP value for C–F.

The C–F cleavage of FLO on Pd(111)/B–C and Pd(111)/C, considering both no H* and H* preadsorbed conditions, was subsequently computed to explore the effects of B on FLO defluorination kinetics. The activation energies (*E*_a_) of the C–F cleavage reactions were lower on Pd(111)/B–C (no H*: 1.96 eV, with H*: 1.85 eV) as compared to those on Pd(111)/C (no H*: 2.20 eV, with H*: 2.12 eV) for both conditions (Figure 4e,f). This supports the faster defluorination kinetics on Pd(111)/B–C. Notably, although H* can effectively reduce the *E*_a_ for C–F cleavage, the *E*_a_ for defluorination on Pd(111)/C for H* preadsorbed condition is still higher than that on Pd(111)/B–C without H* adsorption. This implies that B modification may endow Pd catalysts with H*-free direct C–F cleavage ability in addition to the primarily adopted H*-mediated hydrodefluorination mechanism.^63^ The proposed FLO degradation pathway and related discussion are provided in Text S7 and Figure S24.

#### Key Role of Boron

**Figure 5:**
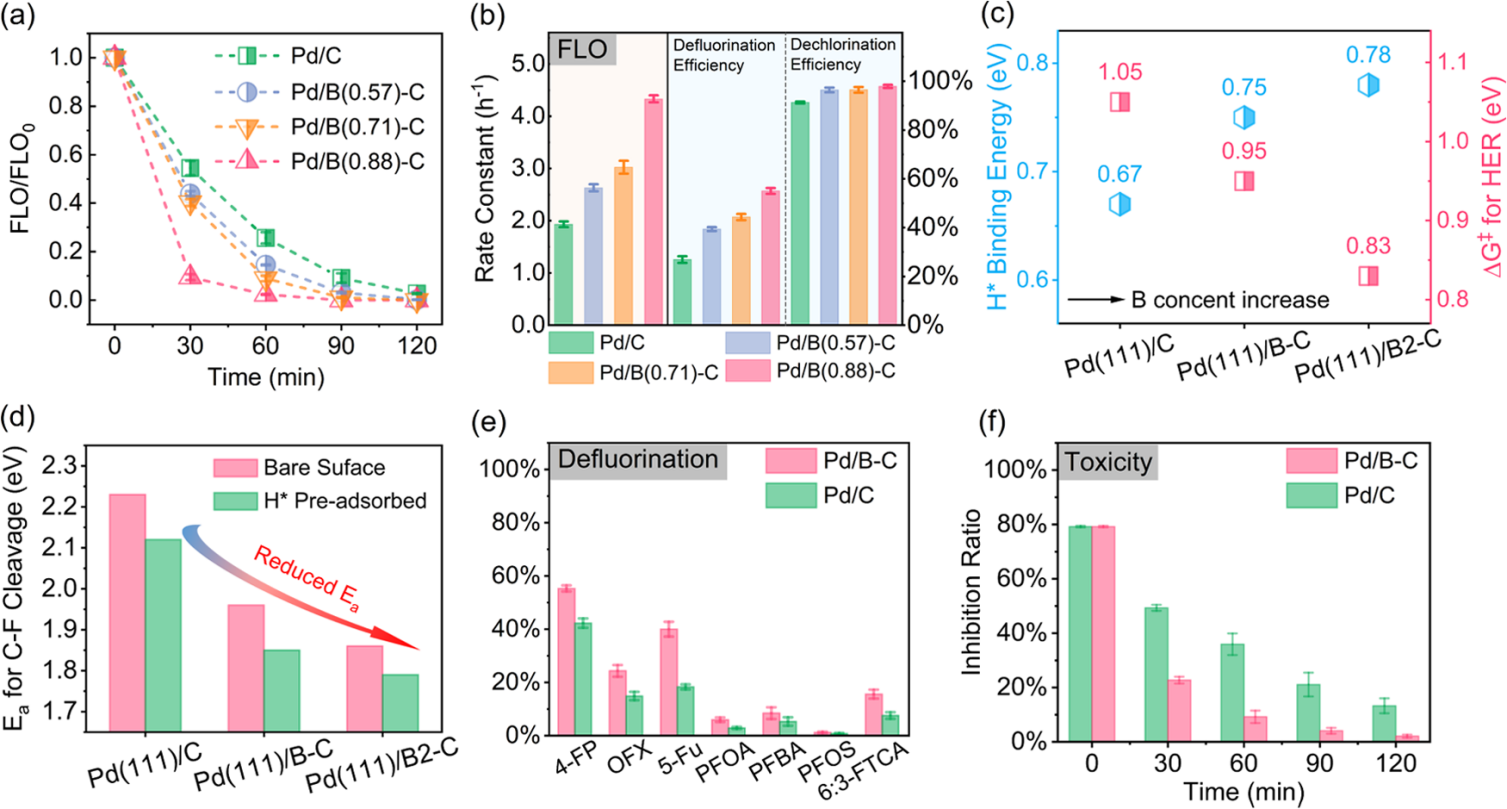
(a) FLO degradation on cathodes with different B contents. (b) FLO degradation rate constants and dehalogenation efficiencies on cathodes with different B contents. (c) DFT simulated H* binding energy and HER Δ*G*^‡^ on cathodes with different B content. (d) DFT simulated FLO defluorination *E*_a_ on bare and H* preadsorbed cathodes with different B content. (e) The defluorination of seven fluorinated compounds on Pd/C and Pd/B–C. (f) Luminescence inhibition ratio for FLO dehalogenation products on Pd/C and Pd/B–C. Conditions: 0.1 M Na_2_SO_4_ solution; *C*_0_ = 20 mg L^–1^; – 1.0 V vs SHE. Data are presented as mean ± s.d. (*n* = 3).

To further explore the crucial role of B, we fabricated Pd cathodes with two other B content (i.e., 0.58 and 0.71 wt % B), denoted as Pd/B(0.58)-C and Pd/B(0.71)-C, respectively, and compared their FLO dehalogenation performance with those of Pd/B(0.88)-C (i.e., Pd/B–C) and Pd/C. As presented, FLO degradation rate constants and dehalogenation efficiencies both increase with B content (Figures 5a,b and S25). The ESR spectra also demonstrate that the generation of H* (i.e., the dominant dehalogenation agent) increases with the B content (Figure S26). DFT calculations were subsequently performed to gain theoretical insights into the crucial role of the B content.

One additional structure, containing twice the number of B atoms as in Pd(111)/B–C, was constructed (denoted as Pd(111)/B2–C). The related optimized structures are provided in Figures S27–S29. The increased H* binding energies with B content imply a better ability to retain H* on cathode surfaces (Figure 5c and Table S11). Additionally, H_2_O dissociation reactions exhibit lower Δ*G*^‡^ values for cathodes with higher B content (Figure 5c and Table S12), suggesting enhanced H* production. The *E*_a_ for FLO defluorination also decreases with the B content on both no H* adsorbed and H* preadsorbed surfaces (Figure 5d and Table S13), implying the crucial role of B on defluorination enhancement. Notably, even though fewer electrons are transferred to the adsorbed FLO after the increase of the B content (Table S14), the distance of Pd–F and *E*_a_ of C–F cleavage still drops. This may be attributed to a more facile activation of C–F stemming from the larger Pd–Pd distance of surface Pd atoms (i.e., stronger strain effect) (Table S10),^46,49,64^ which also correlates with the smaller angles of major diffraction peaks for cathodes with a higher B content (Figure S30). Collectively, increasing the B content can effectively promote the generation and preservation of H* and induce a stronger strain effect for a boosted FLO defluorination kinetic. This is consistent with previous studies, which show that catalysts with higher B doping levels generally exhibited improved catalytic performance (e.g., O_2_ reduction to H_2_O_2_^65^ and H_2_ production^40^). Accordingly, future studies focused on optimizing catalyst fabrication methods to increase the B content are encouraged for further enhanced defluorination kinetics. However, it is important to note that increasing the B content may lead to higher costs or potential mechanical instability of catalysts. As such, fabrication methods should be carefully optimized to achieve an optimal B content that ensures the best overall performance.

### Defluorination of Other Fluorinated Compounds

To explore the general applicability of Pd/B–C for defluorination, seven additional fluorinated compounds, namely, 4-fluorophenol (4-FP), ofloxacin (OFX), 5-fluorouracil (5-Fu), perfluorobutanoic acid (PFBA), perfluorooctanesulfonic acid (PFOS), (2*H*,2*H*,3*H*,3*H*)-perfluorononanoic acid (6:3-FTCA), and perfluorooctanoic acid (PFOA) were selected as the target pollutants to further validate the defluorination performance of Pd/B–C. These compounds are all widely detected in water matrices and cover various types of C–F bonds or fluorinated structures including C–F bonds in aromatic bonds, C = C bonds, and perfluoroalkyl substances (PFAS). As shown in Figure 5e, Pd/B–C demonstrates defluorination efficiencies that are 30.8–118.9% higher than those of Pd/C for all seven fluorinated compounds. This suggests the general applicability of Pd/B–C as a superior cathode material for the defluorination of various types of fluorinated compounds. The low defluorination efficiencies of PFOA and PFOS may be attributed to the strong binding of the C–F bond, resulting from their low one-electron reduction potential and their nature of negative charge because of low p*K*_a_ (<0).^66^ Generally, the negatively charged PFOA can barely reach the cathode surface owing to electrostatic repulsion, which would result in low PFOA degradation efficiency.

### Toxicity, Durability, and Practical Applicability Assessment

The inhibition ratio for luminescent bacteria *V. fischeri* was employed as an indicator for antimicrobial activity and toxicity during electrocatalytic FLO reduction. After electrolysis, the remaining inhibition ratio for the Pd/B–C-treated solution (2.1%) is lower than one-sixth that of the Pd/C-treated one (13.3%) (Figure 5f). This confirms the higher performance in reducing the antibacterial activity and toxicity for Pd/B–C.

Sustaining robust durability is crucial for materials in practical applications. Throughout 10 cycles of repeating experiments (120 min each), Pd/B–C maintains stable efficiencies for FLO destruction and dehalogenation (Figure S31). The FLO destruction, dechlorination, and defluorination efficiencies in the tenth cycle still reach up to 98.5, 90.6, and 83.5%, respectively, as those in the first cycle. This suggests prominent stability and reusability of Pd/B–C. Additionally, the minimal Pd release further confirms the exceptional stability (Figure S32). The increase of the Pd^0^ content in the XPS spectra after use also suggests the good durability of Pd/B–C (Figure S33).

The effects of both Cl^–^ (50, 100, and 200 mg L^–1^) and humic acid (HA) (1, 5, and 10 mg L^–1^) on ER processes were also evaluated because of their wide distribution and known scavenging effects. Only minimal inhibition was observed, even at the highest concentrations of Cl^–^ and HA tested (Figures S34 and S35). Additionally, the slightly reduced FLO degradation performance in the presence of other common ions (NO_3_^_–_^, HCO_3_^_–_^, and CO_3_^2–^) and under O_2_-saturated conditions further supports the robustness of Pd/B–C (Figures S19 and S36). These findings suggest that Pd/B–C demonstrates a strong stability and feasibility for practical applications. To further validate this deduction, FLO destruction by Pd/B–C in real-water matrices (tap water and river water), which were spiked with 20 mg L^–1^ FLO, was tested. The water quality parameters are summarized in Table S15. After 60 min, the FLO destruction efficiencies in tap water and river water are only 0.6 and 5.9% lower than that in 0.1 M Na_2_SO_4_ DI water (Figure S37), respectively. The preservation of most FLO destruction efficiencies in real-water matrices indicates that Pd/B–C is a suitable cathode material for FLO destruction in practical applications. Additionally, the remarkably low energy consumption, with an electric energy per order (EEO) of 0.20 kWh m^–3^ for Pd/B–C, compared to other FLO degradation processes, further highlights the practical application potential (Table S16).

### Environmental Implications

ER is a clean and efficient technology to reduce the biotoxicity of halogenated antibiotics by breaking carbon halogen bonds. However, detoxification is largely hindered by the limited defluorination efficiency because of the high BDE of C–F bonds. This work presents an effective cathode design strategy to enhance C–F cleavage by loading benchmark Pd cathodes onto B–C. Distinct from the typical H*-mediated process, B doping improves the intrinsic C–F breakage capability of the Pd cathode, as evidenced by the exclusive formation of direct C–F cleavage products (i.e., FLO-F) on Pd/B–C, leading to significantly improved defluorination and detoxification efficiencies of various fluorinated compounds. The increased electron density and enhanced strain effects on surface Pd atoms, induced by B doping, are identified as critical factors to reduce the energy barrier for C–F cleavage and remarkably enhanced defluorination. More importantly, these insightful findings could inspire us to advance the cathode design that is tailored for more efficient defluorination by strengthening these two factors. Collectively, this study sheds light on cathode design strategies for effective C–F bond cleavage, paving the way for the efficient destruction and detoxification of fluorinated organic compounds in aquatic systems.

## Supporting Information Available

Chemicals and materials; experimental setups; theoretical calculation methods; effects of various factors on FLO degradation; degradation by-products; and additional DFT calculation results (PDF)
