# Supplementary material for: B-Modified Pd Cathodes for the Efficient Detoxification of Halogenated Antibiotics: Enhancing C–F Bond Breakage beyond Hydrodefluorination
Source: Environ Sci Technol. 2025 Mar 11;59(11):5808–18. doi: 10.1021/acs.est.4c12635 (PMC11948477; doi:10.1021/acs.est.4c12635)
Supplement: Supplementary file 1 — es4c12635_si_001.pdf [file es4c12635_si_001.pdf]

Supplementary Information for

B-modified Pd cathodes for efficient detoxification of halogenated antibiotics: Enhancing C-F bond breakage beyond hydrodefluorination

Zefang Chen[a], Lin Du[b], Victor Fung[c*], Qingquan Ma[a], Xiaojun Wang[b*], Shaohua Chen[b], John C. Crittenden[a, d], and Yongsheng Chen[a*]

[a] School of Civil and Environmental Engineering, Georgia Institute of Technology, Atlanta, GA 30332, United States

[b] CAS Key Laboratory of Urban Pollutant Conversion, Institute of Urban Environment, Chinese Academy of Sciences, Xiamen 361021, P.R. China

[c] School of Computational Science and Engineering, Georgia Institute of Technology, Atlanta, GA 30332, United States

[d] Crittenden and Associates, Atlanta, Georgia, United States

Corresponding authors:

*Victor Fung, Xiaojun Wang, and Yongsheng Chen

Email: victorfung@gatech.edu; xjwang@iue.ac.cn; yongsheng.chen@ce.gatech.edu

This PDF file includes:

Text S1 to S7

Figures S1 to S38

Tables S1 to S16

SI References

Supporting Information Text

Text S1. The information for chemicals and materials

The glassy carbon electrode (ϕ 5 mm) and the Hg/Hg_2SO_4 reference electrode (0.652 V vs. standard hydrogen electrode (SHE)) were purchased from Gaoss Union. The carbon cloth (W0S1011) was acquired from Cetech Co.,Ltd. The graphite rod electrode (ϕ 6 mm*120 mm) was purchased from Ledonlab (Shanghai, China). The Nafion N-117 membrane (0.18 mm thick, ≥ 0.9 mequiv/g exchange capacity) and the HPLC grade methanol (≥ 99.9%) were obtained from Thermo Fisher Scientific (Shanghai, China). The FLO was purchased from Macklin (Shanghai, China). The Na_2SO_4 (≥ 99%), potassium dihydrogen phosphate (≥ 99.5%), dipotassium hydrogen phosphate trihydrate (≥ 99.0%), and phosphoric acid (≥ 85.0%) were purchased from Sinopharm Chemical Reagent Co (Shanghai, China). The carbon black was purchased from Tanaka Kikinzoku Kogyo K.K (TKK). The Nafion 117 solution (5 wt %) and 5-Fluorouracil (≥ 99%) were acquired from Sigma-Aldrich (Shanghai, China). The tertiary butanol (*t*-BuOH) (≥ 99.5%), the boric acid (≥ 99.5%), the palladium acetate (99.98%), the ofloxacin (98%), the 4-Fluorophenol (99%) and the HPLC grade formic acid (≥ 98%) were acquired from Aladdin (Shanghai, China). The perfluorooctanoic acid (PFOA, 97.0%) was purchased from Acmec Biochemical Co. (Shanghai, China). The 5,5′-Dimethyl-1-pyrroline-oxide (DMPO) was purchased from Coolbjc (Anhui, China). The luminescent bacteria (Vibrio *fischeri*) were purchased from J&Q Environmental Technologies Co., Ltd., (Beijing, China). The commercial Pd/C (ca. 10 wt.%) was acquired from Benze Reagent (Tianjin, China). All experimental solutions were prepared using ultrapure water (> 18.2 MΩ cm at 25±1 °C, hereafter referred as UW),

which was generated by a Millipore Direct-Q5 system. UW was used in all the experiments except otherwise stated. All chemicals used in the experiments were reagent grade or higher and used as received without further purification.

Text S2. The details for XAFS data processing and wavelet data parameters

The XAFS data were processed according to the standard procedures using the Athena module implemented in the IFEFFIT software packages. The EXAFS spectra were obtained by subtracting the post-edge background from the overall absorption and then normalizing with respect to the edge-jump step. Subsequently, the $\chi(k)$ data of were Fourier transformed to real (R) space using a hanning windows (dk = 1.0 Å^{-1}) to separate the EXAFS contributions from different coordination shells. To obtain the quantitative structural parameters around central atoms, least-squares curve parameter fitting was performed using the ARTEMIS module of IFEFFIT software packages [1, 2].

For Wavelet Transform analysis, the $\chi(k)$ exported from Athena was imported into the Hama Fortran code [3]. The parameters were listed as follow: R-range, 0.0−4.0 Å, k-range, 0−16.0 Å^{-1} for sample and Standards; k weight, 2; and Morlet function with κ=8, σ = 1 was used as the mother wavelet to provide the overall distribution.

Text S3. The details for a typical run

In a typical run, 12 mL of 20 mg L^{-1} FLO solution with 0.1 M Na_2SO_4 was added to the cathode chamber equipped with the working electrode, and a constant cathode potential (1.0 V vs. SHE) with IR compensation was controlled using the Amperometric I−T technique. The solution was under an 800-rpm magnetic stirring to maintain a homogeneous state throughout the electrolysis. A control experiment confirms that the

FLO evaporation and adsorption is negligible. Before the electrolysis began, FLO solutions were stirred for 15 min to reach adsorption/desorption equilibrium. All electrolysis experiments were carried out in triplicate at room temperature (25±1 °C). Except for the experiments for different pH, all potentials reported are in reference to the SHE.

Text S4. The details for electrochemical characterizations

The electrochemical impedance spectroscopy (EIS) was obtained using the IMP-A.C. Impedance technique with an amplitude of 5 mV and a frequency range of 100 k to 10 Hz at both the open-circuit potential and the common working potential (−1.0 V vs. SHE). The cyclic voltammograms (CV) were acquired between −1.0 and 0.45 V vs. SHE at 50 mV s^{-1}. The electrochemical double-layer capacitance (Cdl) for the working electrode was estimated by sweeping the cathode potential between 0.45 to 1.15 V vs. SHE and analyzing the capacitive currents at 0.85 V vs. SHE for different sweeping speeds (5, 10, 20, 30, 40, 60, 80, and 100 mV s^{-1}).

Text S5. The methods for DFT simulations

Text S5.1. The details for conformer search

For the conformer search, Confab was first employed to classify ~60000 possible conformers into 1249 similar structures [4]. Crest was then employed to invoke xtb for batch geometry optimization and energy calculation of the 1249 structures by the GFN2-xTB method under implicit solvation model [5-8]. Afterwards, Molclus was applied to refine the 1249 structures into 98 conformers based on geometry and energy similarity [9]. Finally, Gaussian 16 was further employed to perform geometry optimization by B3LYP-D3(BJ)/6-31G(d,p) and single point energy calculation by M062x-D3(0)/def2TZVPP of

the top 15 conformers with lowest energies [10-18]. The conformer with the lowest Gibbs free energy was the preferred one.

Text S5.2. The details for VASP calculations

The generalized gradient approximation (GGA) with the functional described by Perdew–Burke–Ernzerhof (PBE) functional was used for structure optimization [19]. The projector augmented wave (PAW) method was used to describe the wave function of the core region [20], while the valence wave function was extended to a linear combination of plane waves with 400 eV cut-off energy.

Two-dimensional single-layer graphene (denoted as C) and boron-doped graphene (denoted as B-C) were used to represent carbon black (CB) and B-doped CB, respectively, because of the following two reasons. First, the XRD characterization shows that both samples exhibited typical (002) reflections of the graphite hexagonal structure (Fig. S30a) [21]. Second, the high resolution XPS spectra of C 1s and O 1s of both samples are characteristic of pristine graphene (Fig. S38) [22].

Since Pd-O coordination is absent in both Pd/B-C and Pd/C, as suggested by the results of EXAFS (Table S3), Pd nanoparticles are likely to reside on C and B-C without oxygen species present. Accordingly, heterostructures were constructed for pure Pd on C or B-C. Pd(111) was selected as the exposed facet because it exhibits a higher activity compared to other facets [23]. The constructed heterostructures are denoted as Pd(111)/C and Pd(111)/B-C, respectively. To explore the effects of B content, we constructed an additional heterostructure with twice as much boron content in Pd(111)/B-C, and the resulted heterostructure is denoted as Pd(111)/B2-C. To calculate binding energies between Pd(111) and C or B-C and the related charge density difference analysis, we used

Pd(111) slabs with three atomic layers in the hexagonal 2 × 2 supercells on C or B-C with equivalent size. The Brillouin zone integration was performed using a 5 × 5 × 1 k-mesh with the Γ-centered scheme. One carbon atom, which is aligned to the face-centered-cubic (fcc) site of Pd(111), was changed to boron to represent B-C because of a high binding energy with Pd(111) compared to that aligned to the hexagonal close-packed (HCP) site. To compute HER reactions, we used Pd(111) slabs with three atomic layers in the hexagonal 4 × 4 supercells with the bottom two Pd layers fixed on C or B-C with equivalent size. The Brillouin zone integration was performed using a 3 × 3 × 1 k-mesh with Γ-centered scheme. To compute H* binding energies, FLO defluorination reactions, and other related electronic property calculations, we used Pd(111) slabs with three atomic layers in the hexagonal 6 × 6 supercells with the bottom two Pd layers fixed on C or B-C with equivalent size. The Brillouin zone integration was performed using a 2 × 2 × 1 k-mesh with Γ-centered scheme.

The vacuum thickness for all heterostructures was set as 25 Å, and the spin-polarized method was applied in all calculations. The weak Van der Waals interaction was described by dispersion correction PBE + D3 [20]. In geometric optimization, the total energy was converged to 10^{-5} eV, and the Hellmann-Feynman force on each relaxed atom was less than 0.03 eV/Å. Transition state (TS) searches were performed by climbing image nudged elastic band (CI-NEB) method [24]. All thermodynamic properties were further acquired using Vaspkit after frequency calculation [25]. Bader charge was obtained using the code from Henkelman group [26]. Binding energies (E_b) were estimated using Eq. S1.

$$E_b = E_T - E_1 - E_2 \tag{S1}$$

Where, E_T, E_1, and E_2 are the total electronic energies of the bonded structure, fragment one, and fragment two, respectively. The structures of fragments one and two were set as the same as in the bonded structures.

Text S5.3. The details for Gaussian calculations

The Becke 3-parameter-Lee-Yang-Parr (B3LYP) functional combined with the 6-31G(d,p) basis set was used for geometry optimizations and frequency calculations [17, 18]. The scale factor of 0.9838 was used to correct for zero-point energy (ZPE) calculations using Shermo software that was developed by Lu et al [27, 28]. As for single point energy calculation, the M06-2X functional was employed because of the excellent performance on main-group thermochemistry, especially on organic species [11]. The def2-TZVPPD basis set was employed for single point energy calculations when estimating the standard reduction potential (E^0) of FLO/FLO^- [16, 29]. The definition of def2-TZVPPD basis set was acquired from the BSE database [30-32]. For other calculations, the def2-TZVPP basis set was used for single point energy calculation [15, 16]. For all calculations, the D3 dispersion (i.e., D3(0) for M06-2X and D3(BJ) for B3LYP) was considered for better simulation of intramolecular and intermolecular weak interaction; the SMD model was employed to account for the implicit water solvation [33].

The Gibbs free energy (G, kJ/mol) for molecules in the aqueous solution was calculated via Eq. S2.

$$G_{SMD} = G_g + \Delta G_{solv}^{SMD} + 7.91 \quad \text{(S2)}$$

Where, G_g (kJ/mol) and G_{SMD} (kJ/mol) are the G for molecules in the gas phase and in the aqueous solution, respectively, ΔG_{solv}^{SMD} (kJ/mol) is the solvation energy calculated by

SMD model without considering the phase change, and 7.91 (kJ/mol) is the phase change energy from gas standard phase (1 atm) to liquid standard phase (1 M) [34].

ΔG_{solv}^{SMD} (kJ/mol) was calculated according to Eq. S3.

$$\Delta G_{solv}^{SMD} = G_{sol}^{1M} - G_{gas}^{1M} \quad \text{(S3)}$$

Where, G_{sol}^{1M} (kJ/mol) and G_{gas}^{1M} (kJ/mol) are calculated at M05-2X/6-31g(d) level of theory with and without SMD model, respectively [33].

The standard reduction potential (E^0, V) value for a given direct electron transfer reaction was calculated by Eq. S4.

$$E^0 = -\frac{\Delta G_{O/R}^0}{nF} - E_{abs}^0(\mathrm{SHE}) \quad \text{(S4)}$$

Where, $\Delta G_{O/R}^0$ (kJ/mol) is the standard free energy difference between the reductant and the oxidant in the aqueous solution, F is the Faraday constant (96485 C/mol), n is the number of electrons transferred, and $E_{abs}^0(\mathrm{SHE})$ is a reference value for the absolute standard reduction potential of the standard hydrogen electrode (SHE, $E_{abs}^0(\mathrm{SHE})$ = 4.28 V for the SMD model) [35-37].

All calculated thermodynamic values in this work were reported at the standard state of 1 molal (= ~1 M) and 298.15 K except otherwise stated.

Text S6. Effects of various factors on FLO destruction

Text S6.1. Effect of cathode potential

A higher overpotential generally leads to a faster kinetic for electrocatalytic reactions. As expected, the degradation and dehalogenation efficiencies of FLO using Pd/B-C increases as the cathode potential varies from –0.4 to –1.0 V vs. SHE (Fig. S12a). However,

further varying the potential from –1.0 to −1.2 V vs. SHE decreases the efficiencies. The efficiency decrease is attributed to the blocking of the active sites by the excessive bubble generation from the largely enhanced competitive hydrogen generation at high overpotential. Because of the better performance, the cathode potential was maintained at – 1.0 V vs. SHE when evaluating the effects of other factors.

Text S6.2. Effect of solution pH

During FLO electroreduction, the solution pH gradually increases from ~5.7 to ~11.5 for most experiments. Hence, the effects of solution pH on FLO reduction performance were evaluated. As shown in Fig. S12b, when maintaining the potential at –1.0 V vs. SHE, the pseudo first order rate constants gradually increase with pH as pH increases from 2.2±0.1 to 5.8±0.1 and gradually decreases as pH increases further. A plausible explanation for this observation is as follows. When pH is lower than 5.8±0.1, a higher proton concentration at lower pH facilitates both Volmer (i.e., $H^+ + e^- \rightarrow H^*$) and Tafel ($2H^* \rightarrow H_2$) processes, which promotes hydrogen generation but reduces the amount of H* available for FLO reduction. Consequently, a higher pH is beneficial to retain more H* on cathode surface for higher FLO destruction efficiencies. However, when pH is higher than 5.8±0.1, the lower proton concentration will reduce H* generation and thus cause a lower FLO destruction kinetics. Additionally, the point of zero charge (PZC) of Pd/B-C (~7.5) from the zeta potential measurement (Fig. S13) indicates the charge of the cathode surface shifts from positive to negative as pH increases to higher than 7.5. The negatively charged surface will repel the negatively charged FLO (pKa: ~9) molecules when pH > 9 and incur a further lower FLO destruction efficiency at higher pH [38]. Experiments at same cathode potentials versus reversible hydrogen electrode (i.e., –0.6 V vs. RHE) were also conducted

to evaluate pH effects at a comparable hydrogen evolution intensity (Fig. S12c), and the results exhibited a similar trend as pH varied. Collectively, a near neutral pH is beneficial for FLO degradation, suggesting Pd/B-C as a suitable electrocatalyst for practical use.

Text S6.3. Effect of initial FLO concentration

For radical-mediated reactions, higher initial concentrations for parent compounds would generally result in lower quasi steady-state radical (e.g., H*) concentrations and thus reduce pseudo-first order rate constants. Similarly, lower FLO degradation kinetics were observed for higher initial FLO concentrations (Fig. S12d). Notably, when initial FLO concentration increases from 20 to 50 mg L^{-1}, even though the FLO degradation rate constant drops from 4.33 to 2.36 h^{-1}, the defluorination efficiency increases from 52.8% to 69.3% (Table S9). Since the radical-mediated reactions normally have lower rate constants at higher initial pollutant concentrations, the increased defluorination efficiency implies the presence of alternative defluorination mechanisms other than the conventional *H-mediated one on Pd/B-C. FLO molecules may occupy a greater ratio of surface sites at higher initial concentrations, which facilitates the alternative *H-free defluorination mechanism.

Text S7. Proposed FLO Degradation Pathways.

Degradation pathways can generally be deducted by byproduct analysis. For the three FLO degradation byproducts (i.e., FLO-Cl, FLO-2Cl, and FLO-2ClF) shared by Pd/B-C and Pd/C, a similar phenomenon was observed for both Pd/B-C and Pd/C. FLO-Cl concentration first increases to a peak and then decreases, while the concentrations of FLO-2Cl and FLO-2ClF increase over time. This implies that FLO dehalogenation is likely to be initialized by dechlorination, followed by defluorination after complete dechlorination. To support this pathway deduction, we calculated the bond dissociation energies (BDEs) of C-F and C-Cl bonds for FLO and FLO-Cl. For both FLO and FLO-Cl, the BDEs of C-Cl bonds (72.4 and 79.7 kcal/mol) are lower than that of C-F bonds (115.7 and 115.9 kcal/mol). This suggests that C-Cl bonds are easier to break than C-F bonds, complying well with the proposed pathway. For Pd/B-C, the detection of the unique byproduct (i.e., FLO-F) indicates that FLO can also lose fluoride on Pd/B-C before dechlorination. Collectively, the plausible FLO degradation pathways are summarized in Fig. S24.

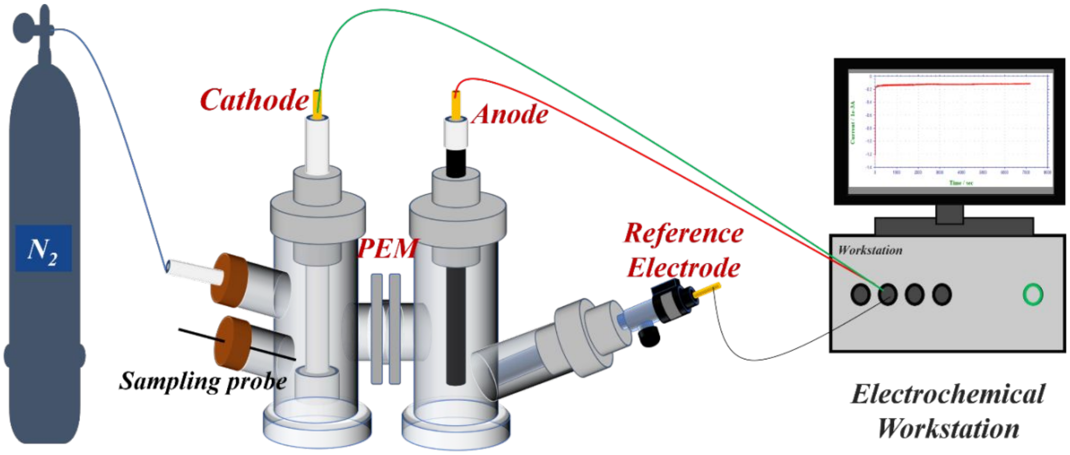

Fig. S1. The schematic of the system for the electrocatalytic FLO reduction.

The (prepared) electrocatalysts (i.e., Pd/B-C, Pd/C, Pd/C (com), and carbon cloth) serve as working electrodes and were put in the cathode chamber. The reference electrode was placed in the anode chamber to minimize the mutual interference between the reference electrode and the working electrode during electrolysis. Similar method was also adopted in the literature [39].

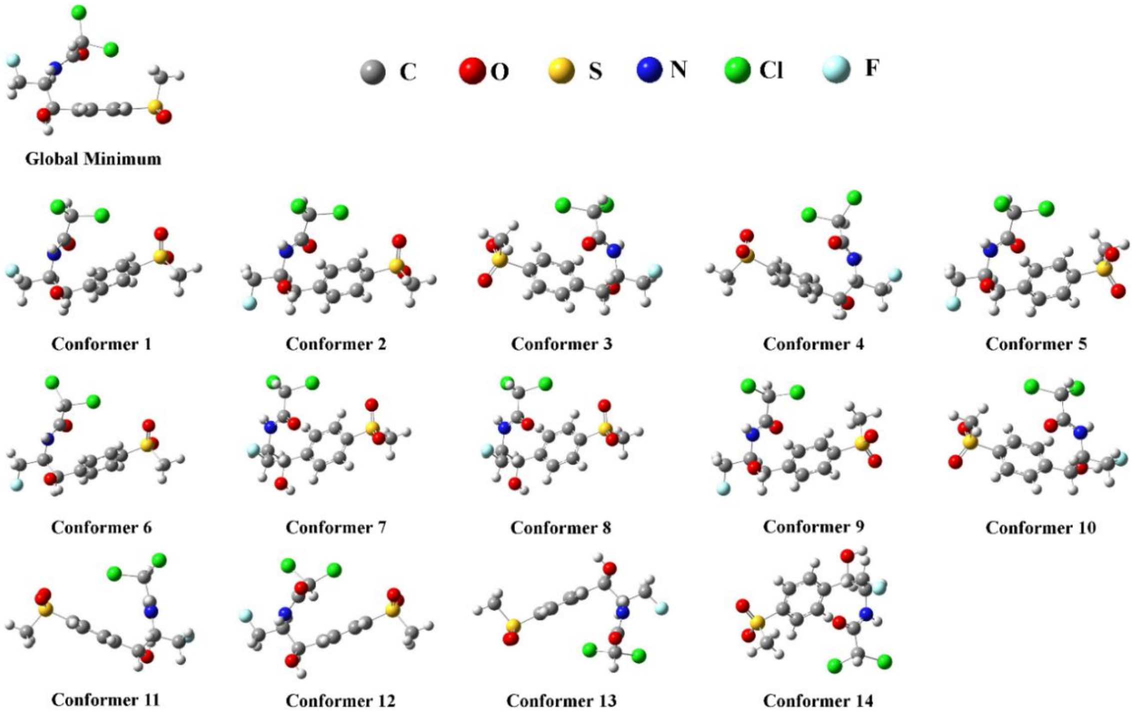

Fig. S2. The optimized structure of FLO and its conformers in the aqueous phase.

The global minimum stands for the optimized structure of FLO, and the rest are the top 14 conformers with the lowest G except for the global minimum.

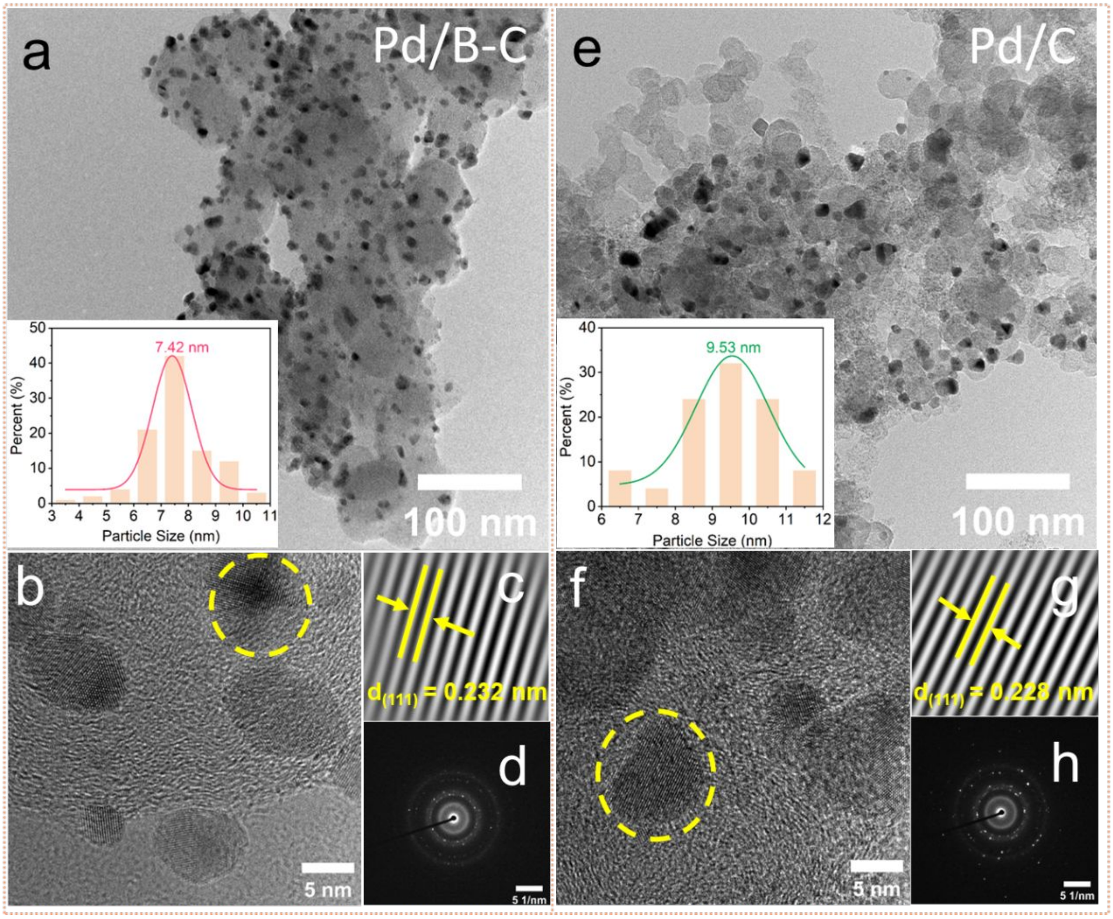

Fig. S3. (a) TEM image and the size distribution histogram (the insert), (b) HRTEM image, (c) Lattice fringe, and (d) Selected area electron diffraction for Pd/B-C. (e) TEM image and the size distribution histograms (the insert), (f) HRTEM image, (g) Lattice fringe, and (h) Selected area electron diffraction for Pd/C. For c and g: $d_{(111)}$ reflects the average distance of several continuous lattice fringes for the nanoparticles that are circled in b and f, respectively.

The selected area electron diffraction (SAED) patterns suggest the same exposed lattices (i.e., (111), (200), (220), and (311) planes of face centered-cubic (fcc) Pd) for both Pd/C and Pd/B-C samples. According to Eq. S6, the percentages of surface Pd atoms for both Pd/B-C and Pd/C are 20.5 and 16.2 %, respectively.

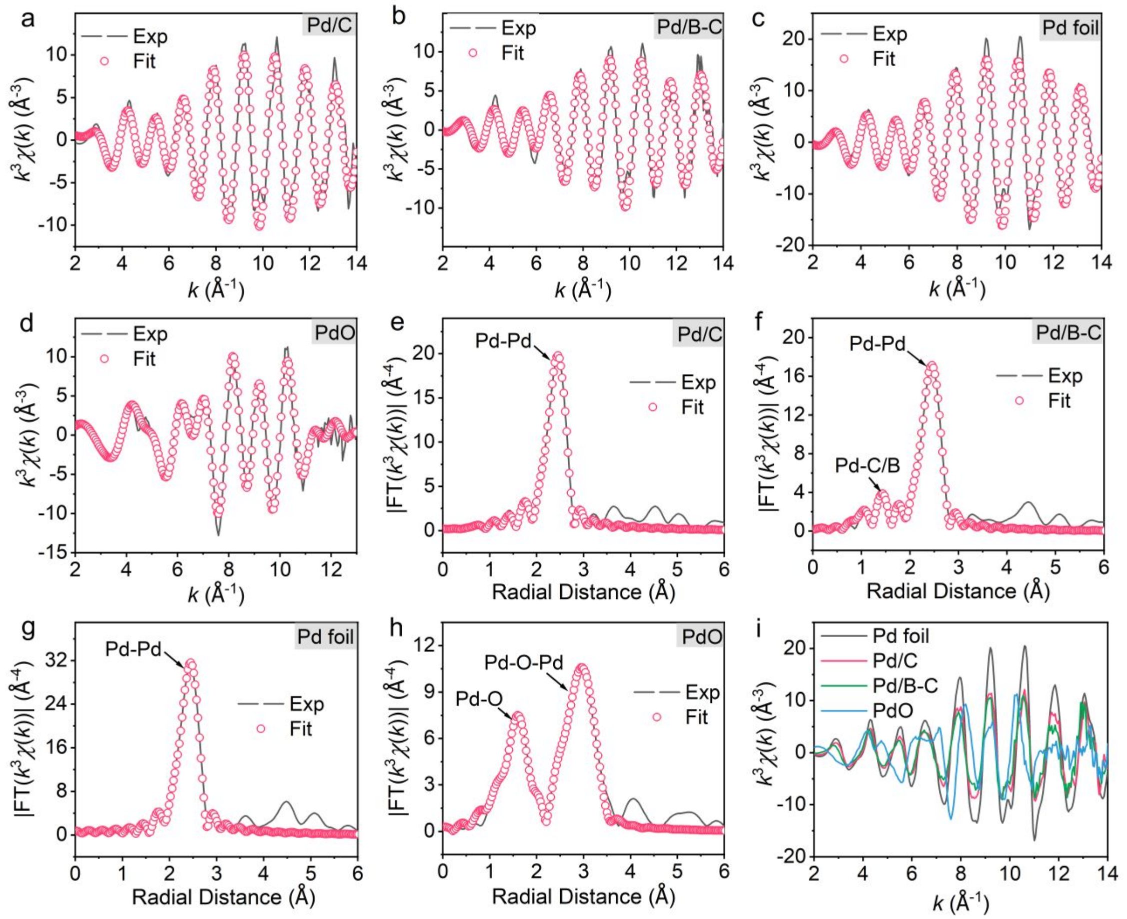

Fig. S4. EXAFS plots of $k^3 .\chi$ experimental and fitted data for (a) Pd/C, (b) Pd/B-C, (c) Pd foil, and (d) PdO. EXAFS plots of $k^3 .\chi$ phase corrected Fourier transform of experimental and fitted data for (e) Pd/C, (f) Pd/B-C, (g) Pd foil, and (h) PdO. (i) EXAFS plots of $k^3 .\chi$ intensity comparison between experimental data for Pd/C, Pd/B-C, Pd foil, and PdO.

The fitted data agrees well with the experimental data. This indicates that the fitting results shown in Table S3 are reliable.

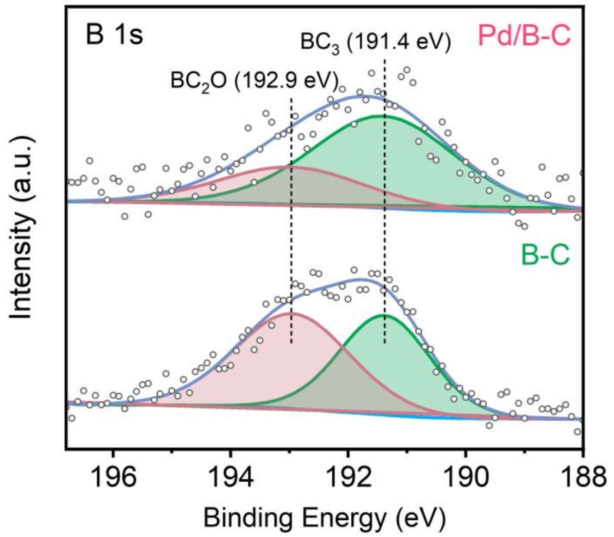

Fig. S5. The high-resolution XPS spectrum of B 1s for freshly prepared Pd/B-C and B-C.

The similar peak locations of the high-resolution B 1s XPS spectra for B-C and Pd/B-C indicate that the charge of B barely changes after loading Pd nanoparticles onto B-C.

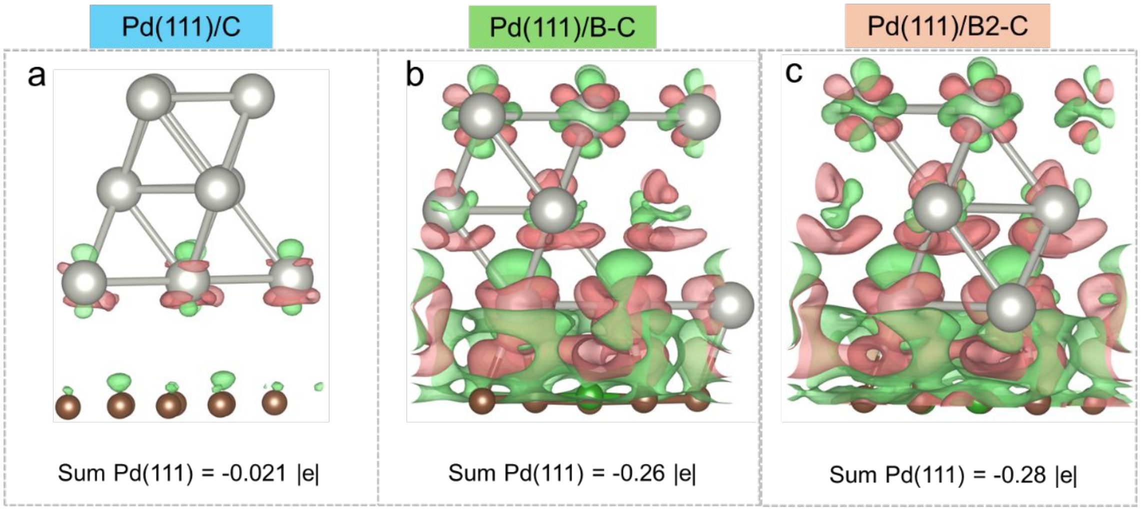

Fig. S6. Charge density difference between Pd(111) and support for (a) Pd(111)/C, (b) Pd(111)/B-C, and (c) Pd(111)/B2-C. Red: charge accumulation. Green: charge depletion. The isosurface value is 0.012 e $Å^{-3}$.

The results of the charge density difference indicate that B doping in the carbon support, as compared to the undoped one, induce more electron transfer from bulk Pd atoms to surface Pd atoms and the carbon supports, and the amount of the transferred electrons increases with B content.

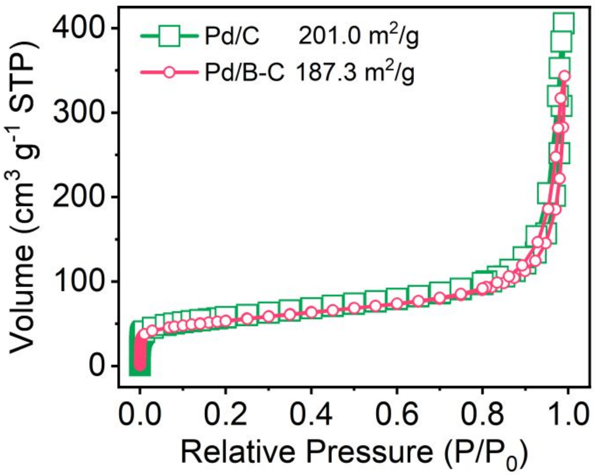

Fig. S7. Nitrogen adsorption/desorption isotherm plot of Pd/B-C and Pd/C.

The similar results of specific surface area (Pd/B-C: 187.3 m^2/g, Pd/C: 201.0 m^2/g) and pore sizes (Pd/B-C: 6.12 nm, Pd/C: 6.20 nm) indicate that the pore structure barely changes after B doping.

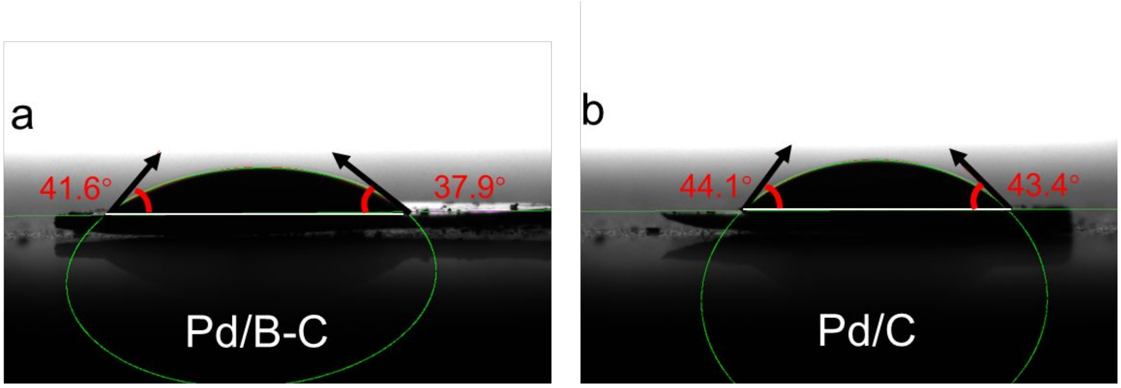

Fig. S8. Contact angle measurements of (a) Pd/B-C and (b) Pd/C.

The contact angles for Pd/B-C (39.8±2.6°) and Pd/C (43.8±0.5°) are close. This indicates that the hydrophobicity of Pd/C barely changes after B doping.

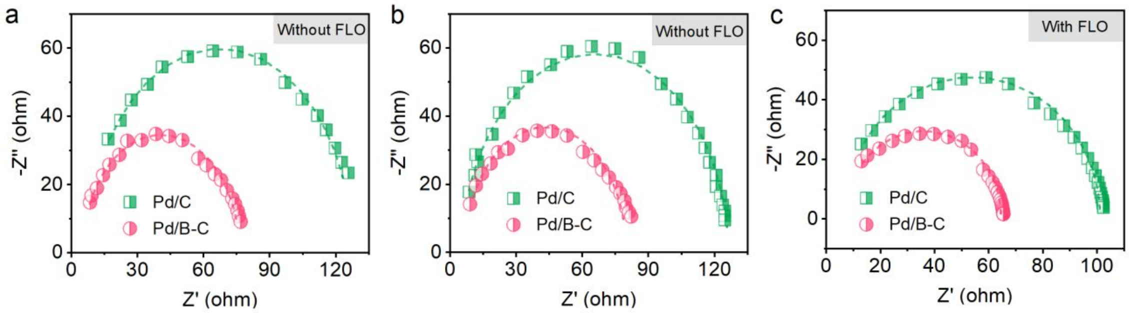

Fig. S9. Nyquist plots of Pd/B-C and Pd/C at (a) –1.0 V vs. SHE, (b) open circuit potential, and (c) open circuit potential. Conditions for (a and b): 0.1 M Na_2SO_4 solution; C_0 = 20 mg L^{-1}; −1.0 V vs. SHE. Conditions for (c): 0.1 M Na_2SO_4 solution; C_0 (FLO) = 20 mg L^{-1}; −1.0 V vs. SHE.

Pd/B-C possesses the lowest charge transfer resistance (R_{ct}) for all conditions (Table S6), implying better electrocatalytic activity for Pd/B-C.

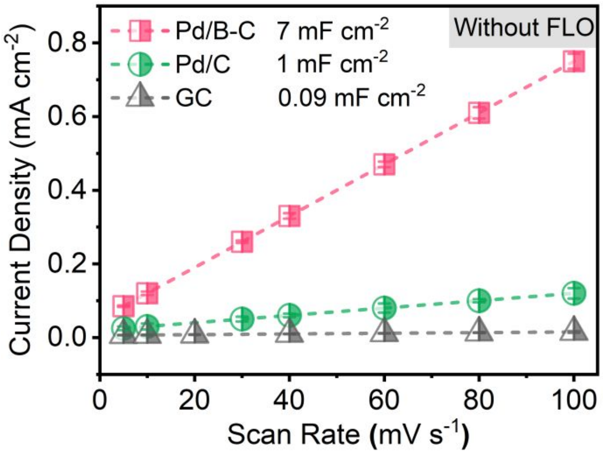

Fig. S10. Linear fitting of the capacitive properties of current density vs. scan rate in a 0.1 M Na_2SO_4 solution.

The electrochemical double-layer capacitance (Cdl) for cathodes is in the order of Pd/B-C > Pd/C > GC. This suggests Pd/B-C has the highest electrochemical active surface area (ECSA) among these three cathodes.

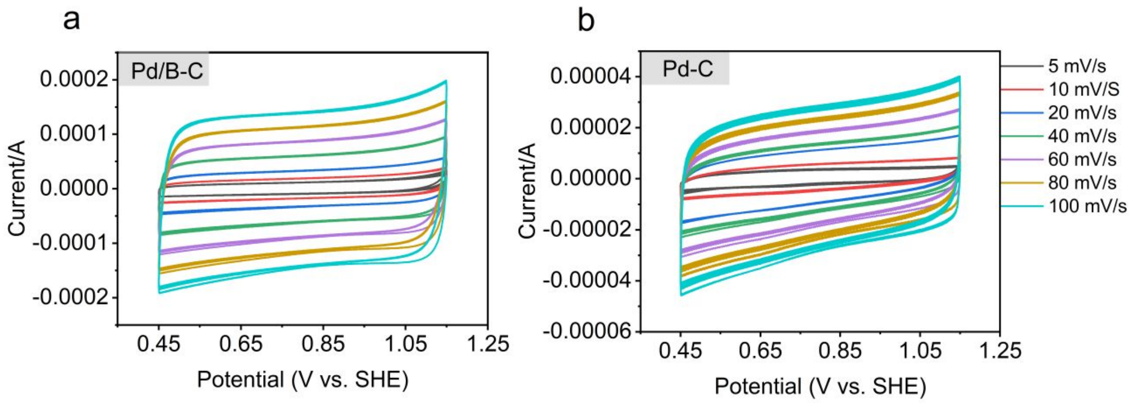

Fig. S11. CV curves of (a) Pd/B-C and (b) Pd/C in the range of 0.45 to 1.15 V vs. SHE. The scan rate is 5, 10, 20, 40, 60, 80, and 100 mV s^{-1} for the curves from inside to outside. Conditions: 0.1 M Na_2SO_4 solution; C_0 (FLO) = 20 mg L^{-1}.

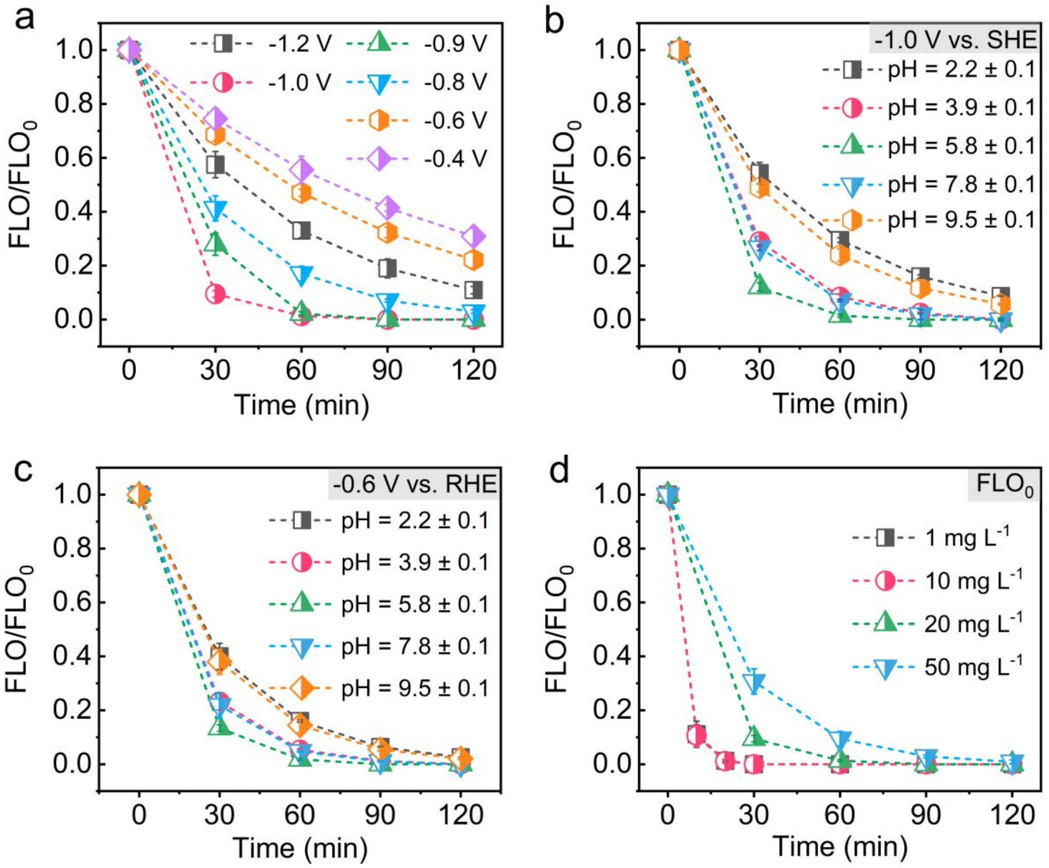

Fig. S12. Effects of (a) cathode potential (C_0 = 20 mg L^{-1}), (b) solution pH at same SHE (C_0 = 20 mg L^{-1}, −1.0 V vs. SHE), (c) solution pH at same RHE (C_0 = 20 mg L^{-1}, −0.6 V vs. RHE), and (d) initial FLO concentration (−1.0 V vs. SHE), on FLO dehalogenation for Pd/B-C. Data are presented as mean ± s.d. (n = 3).

The optimal cathodes potential is −1.0 V vs. SHE. The optimal pH is 5.8±0.1. FLO degradation efficiencies generally decrease with the increase of initial FLO concentration.

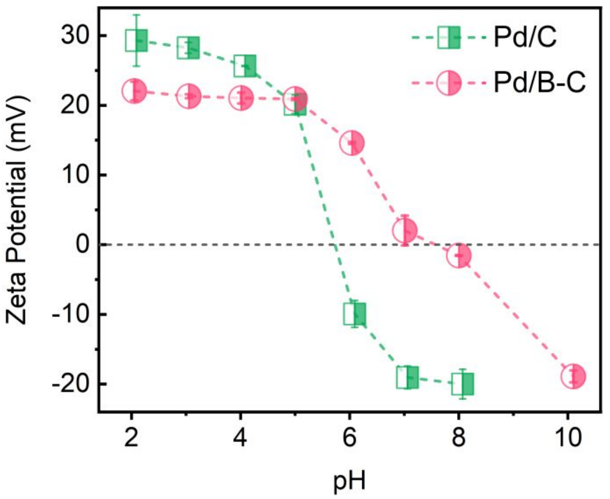

Fig. S13. Zeta potential measurements for Pd/C and Pd/B-C.

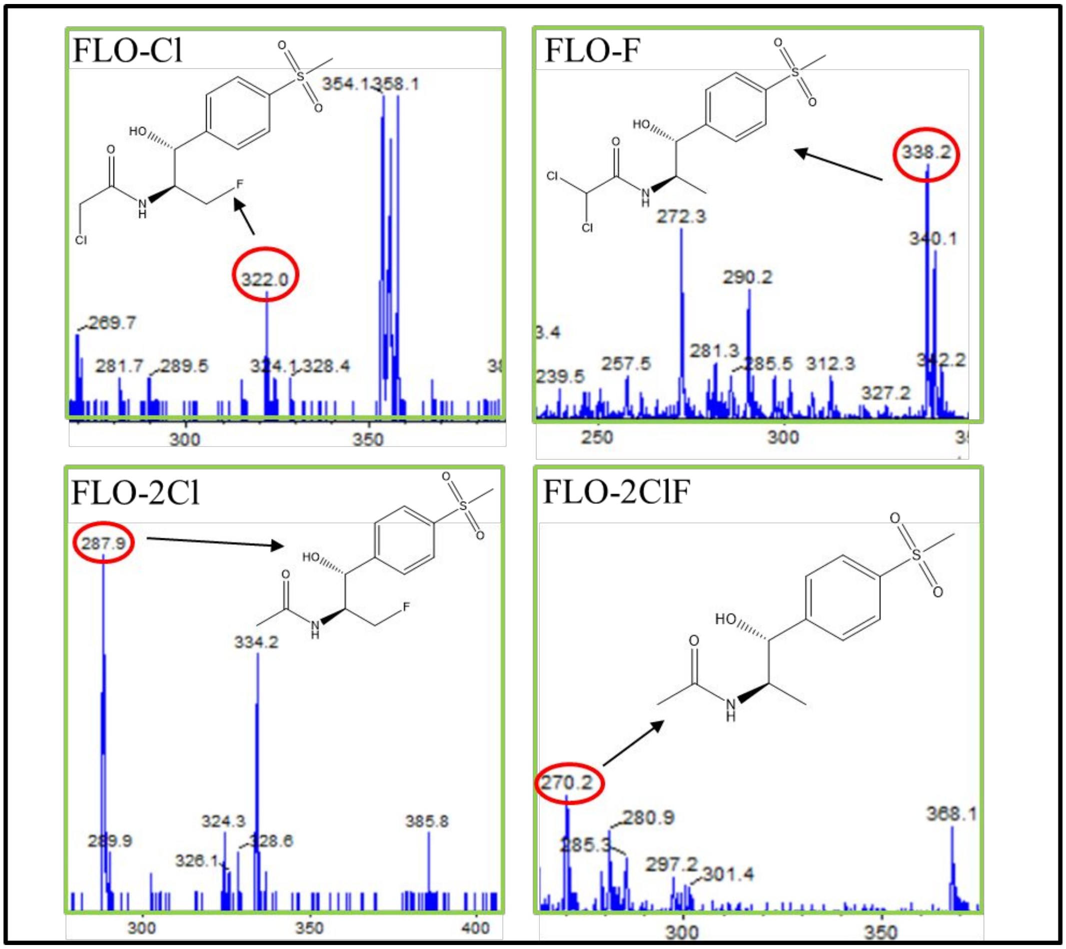

Fig. S14. MS identification of FLO dehalogenation products.

FLO-F was exclusively detected on Pd/B-C, indicating a distinct direct C-F breakage mechanism.

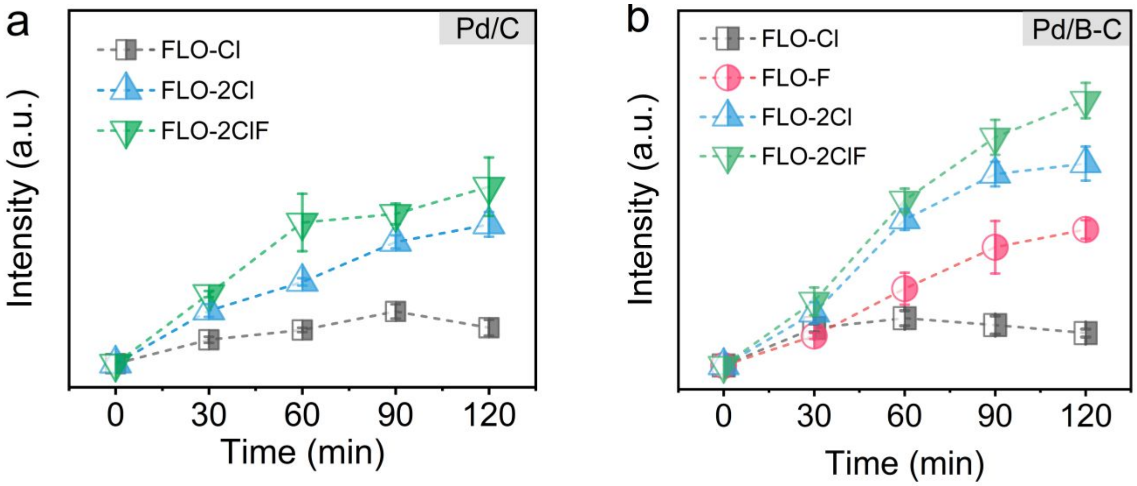

Fig. S15. Semi-quantitative analysis of produced byproducts of electrocatalytic FLO reduction on (a) Pd/C and (b) Pd/B-C cathodes. Conditions: 0.1 M Na_2SO_4 solution; C_0 = 20 mg L^{-1}; −1.0 V vs. SHE; Cathode support: 1 cm^2 carbon cloth; without pH adjustment. Data are presented as mean ± s.d. (n = 3).

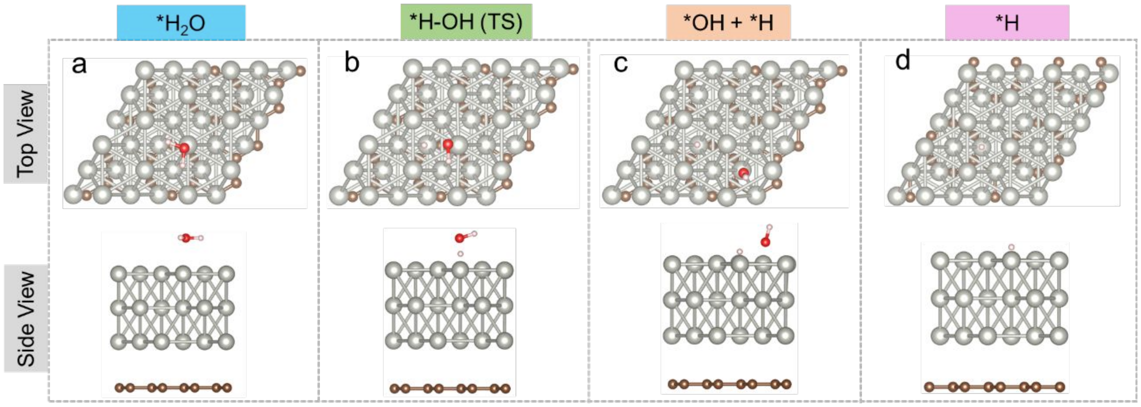

Fig. S16. Top and side view of the optimized configurations of intermediates involved in H_2O dissociation on Pd(111)/C surface.

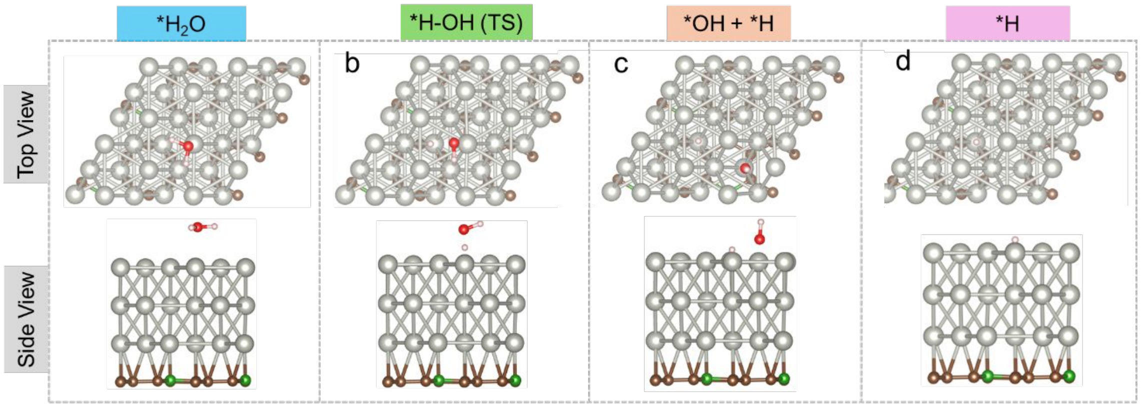

Fig. S17. Top and side view of the optimized configurations of intermediates involved in H_2O dissociation on Pd(111)/B-C surface.

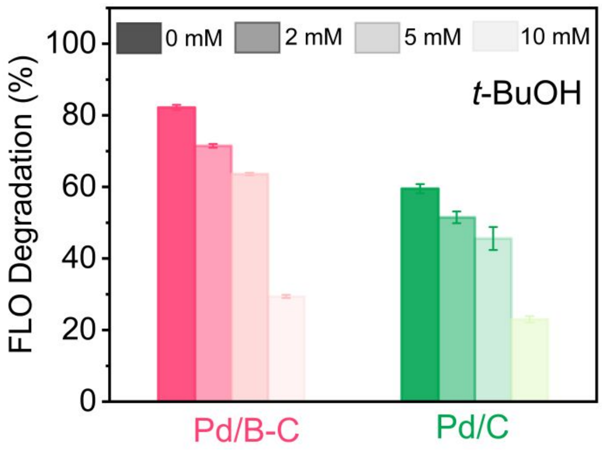

Fig. S18. FLO degradation efficiencies on Pd/B-C and Pd/C under different *t*-BuOH concentrations. Conditions for (d): 0.1 M Na_2SO_4 solution; C_0 = 20 mg L^{-1}; −1.0 V vs. SHE. Cathode support: GC. Data are presented as mean ± s.d. (n = 3).

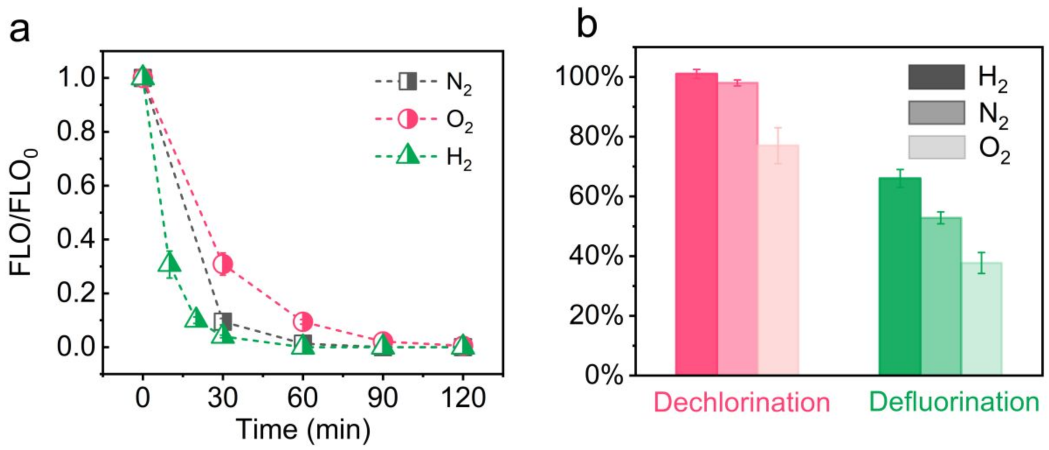

Fig. S19. (a) The degradation and (b) dehalogenation of FLO on Pd/B-C under different atmospheres. Conditions: 0.1 M Na_2SO_4 solution; C_0 = 20 mg L^{-1}; −1.0 V vs. SHE. Cathode support: 1 cm^2 carbon cloth. Data are presented as mean ± s.d. (n = 3).

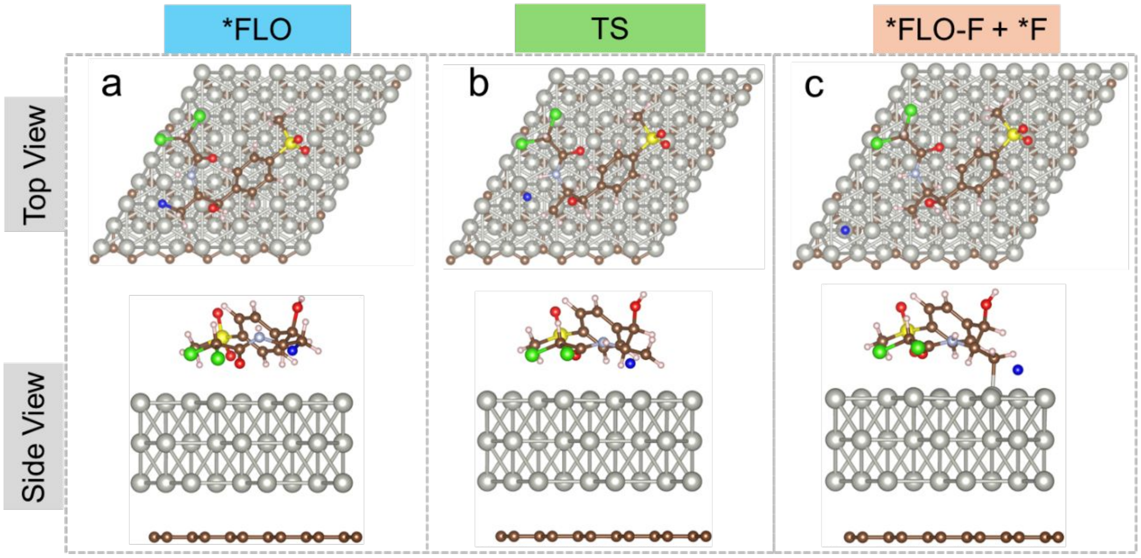

Fig. S20. Top and side view of the optimized configurations of intermediates involved in C-F cleavage of FLO on Pd(111)/C surface.

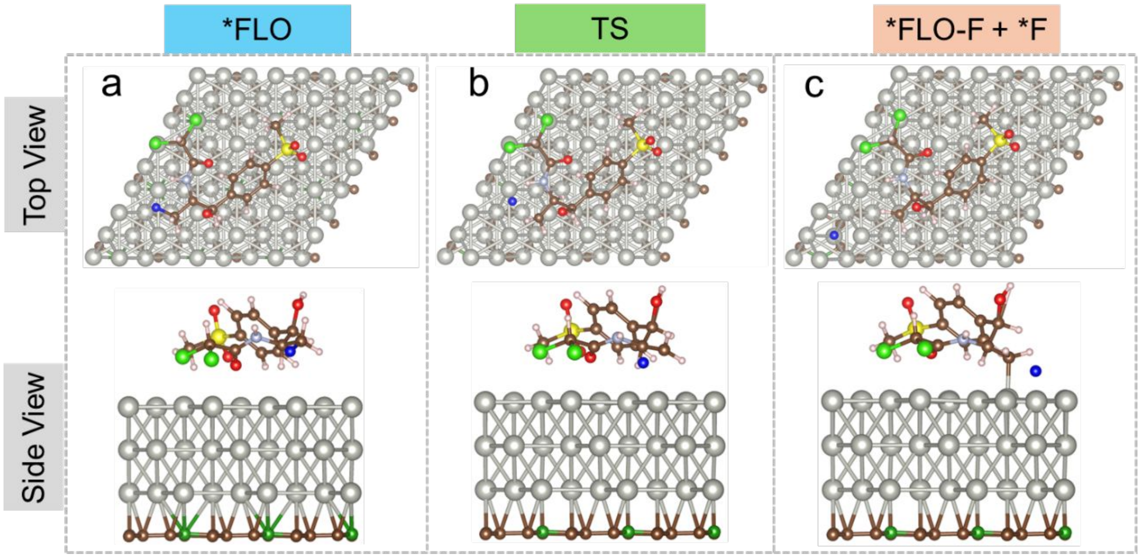

Fig. S21. Top and side view of the optimized configurations of intermediates involved in C-F cleavage of FLO on Pd(111)/B-C surface.

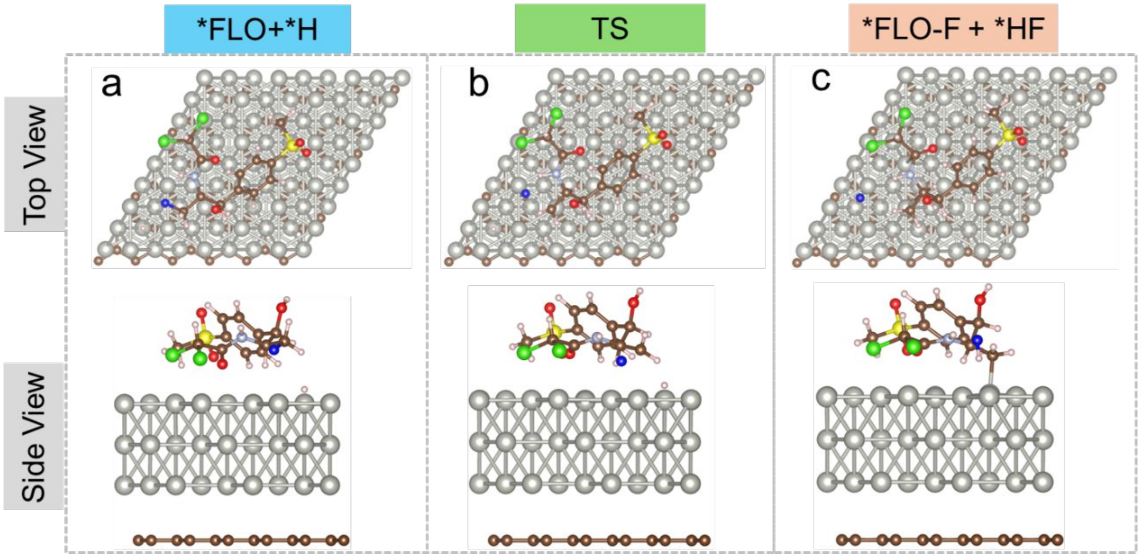

Fig. S22. Top and side view of the optimized configurations of intermediates involved in C-F cleavage of FLO on H* pre-adsorbed Pd(111)/C surface.

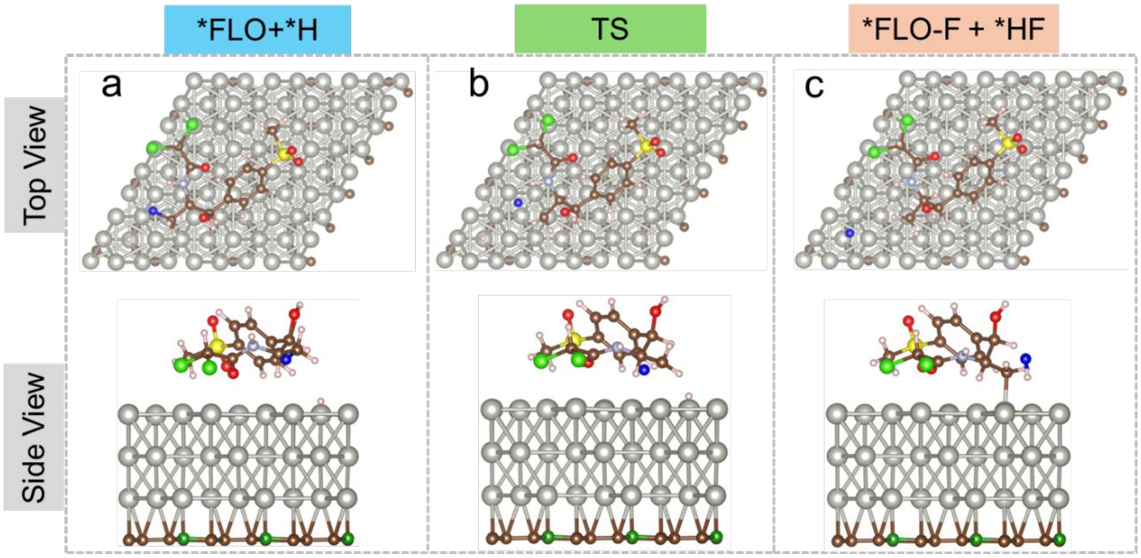

Fig. S23. Top and side view of the optimized configurations of intermediates involved in C-F cleavage of FLO on H* pre-adsorbed Pd(111)/B-C surface.

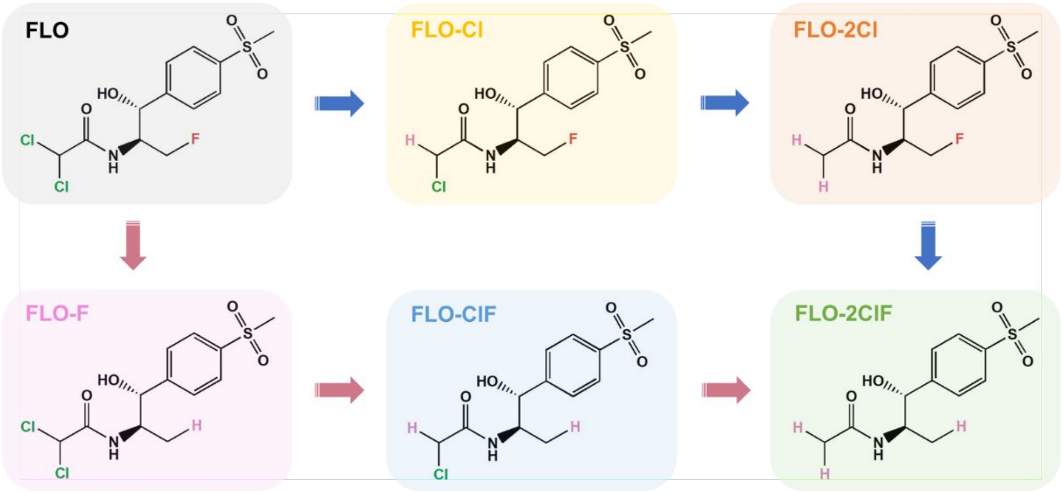

Fig. S24. The plausible FLO degradation pathways. Red arrows represent pathways that are exclusively for Pd/B-C. Blue arrows represent pathways that are shared by both Pd/B-C and Pd/C.

FLO-F is exclusively generated when Pd/B-C was employed as the cathode material.

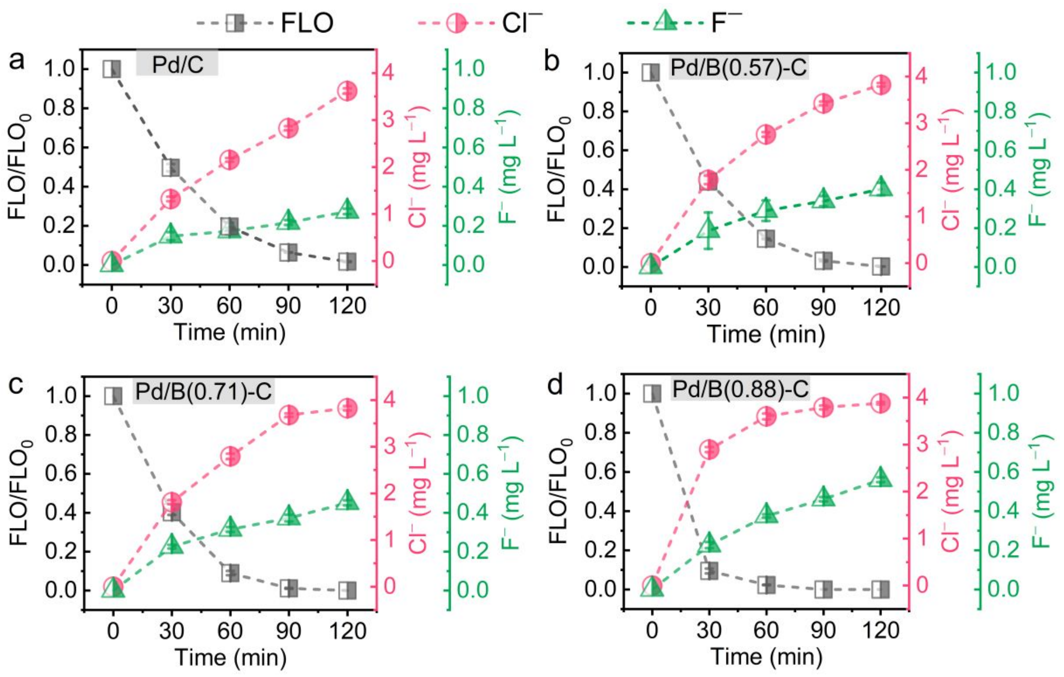

Fig. S25. FLO degradation and released Cl^- and F^- on (a) Pd/C, (b) Pd/B(0.57)-C, (c) Pd/B(0.71)-C, and (d) Pd/B(0.88)-C (i.e., Pd/B-C). Conditions: 0.1 M Na_2SO_4 solution; $C_0 = 20$ mg L^{-1}; −1.0 V vs. SHE. Data are presented as mean ± s.d. (n = 3).

The destruction and dehalogenation kinetics of FLO degradation increase with B content.

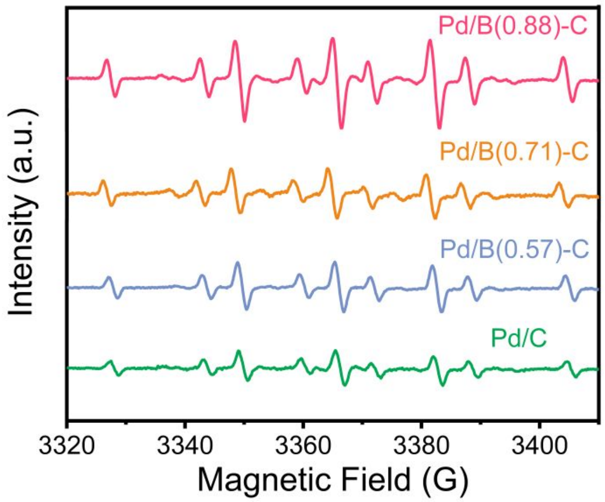

Fig. S26. (c) DMPO spin trapping ESR spectra of cathodes of different B content.

The intensity of DMPO-H peak increases with B content, indicating a higher H* generation at higher B content.

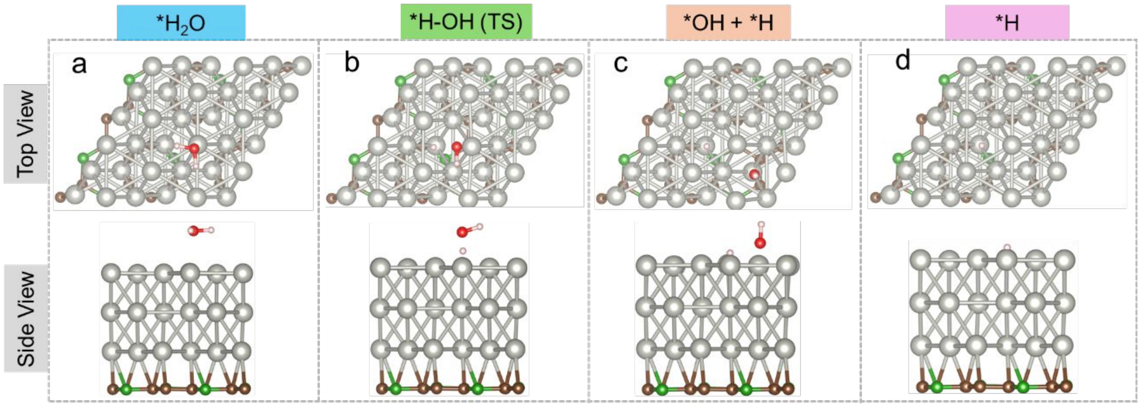

Fig. S27. Top and side view of the optimized configurations of intermediates involved in H_2O dissociation on Pd(111)/B2-C surface.

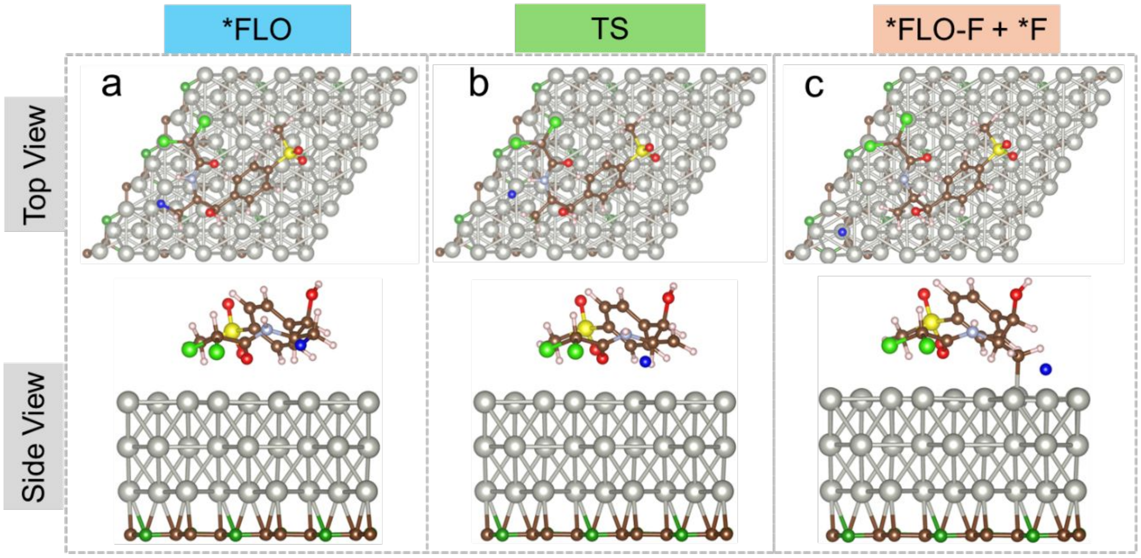

Fig. S28. Top and side view of the optimized configurations of intermediates involved in C-F cleavage of FLO on Pd(111)/B2-C surface.

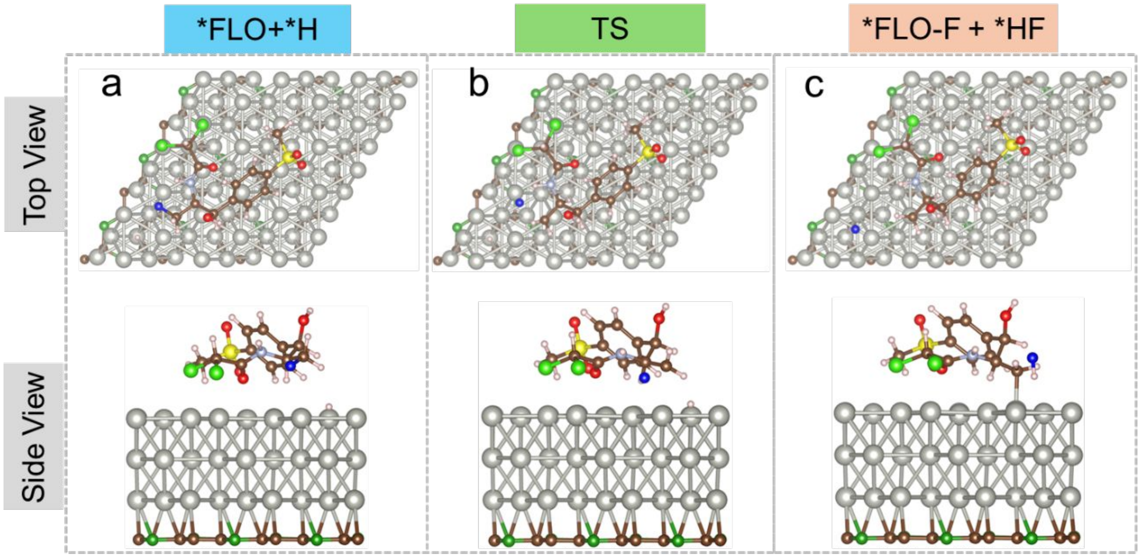

Fig. S29. Top and side view of the optimized configurations of intermediates involved in C-F cleavage of FLO on H* pre-adsorbed boron-modified Pd(111)/B2-C surface.

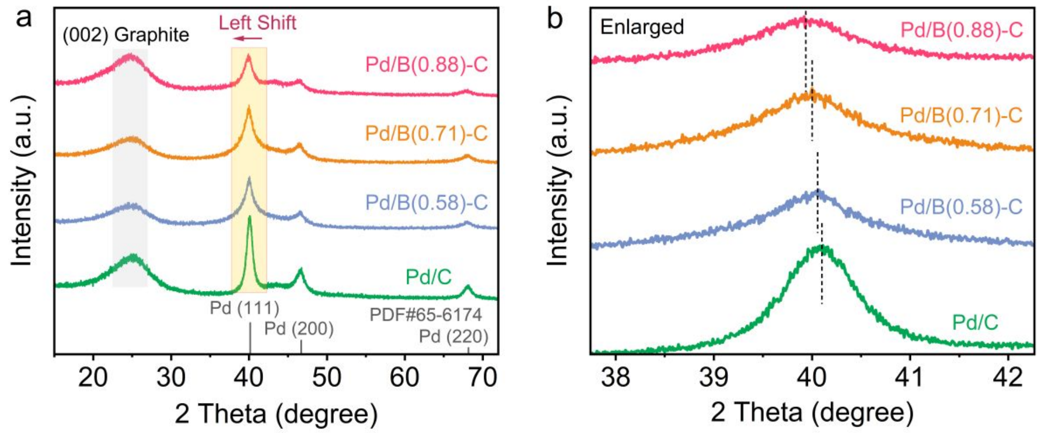

Fig. S30. (a) XRD patterns for freshly prepared cathodes of different B content. (b) Enlarged patterns for Pd(111).

With the increase of B content, the major diffraction peaks for cathodes gradually shift to smaller angles, suggesting the increase of expansion and strain effects of Pd lattice after B doping.

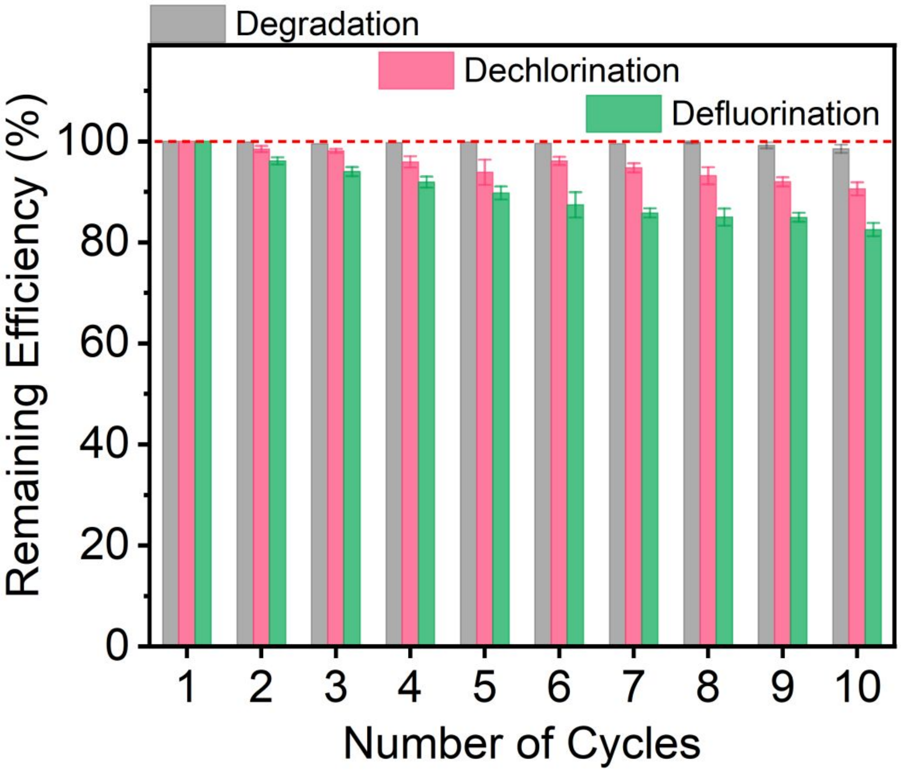

Fig. S31. Reusability of Pd/B-C for FLO degradation and dehalogenation. Data are presented as mean ± s.d. (n = 3).

FLO destruction, dechlorination, and defluorination efficiencies in the tenth cycle still reach up to 98.5, 90.6, and 83.5 % as that in the first cycle, suggesting prominent stability and reusability of Pd/B-C.

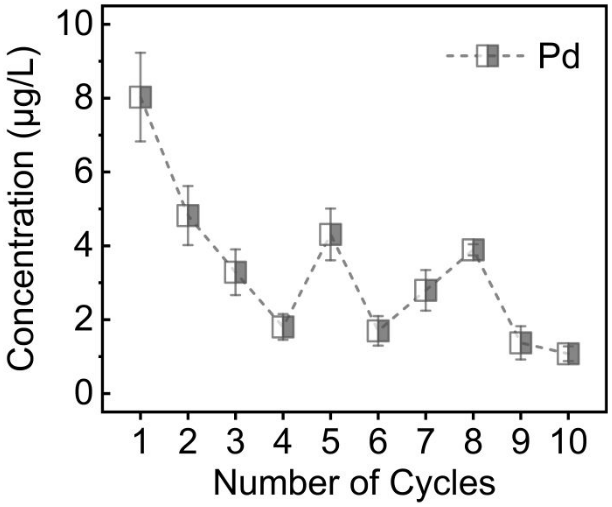

Fig. S32. Leaching of Pd for Pd/B-C at each cycle.

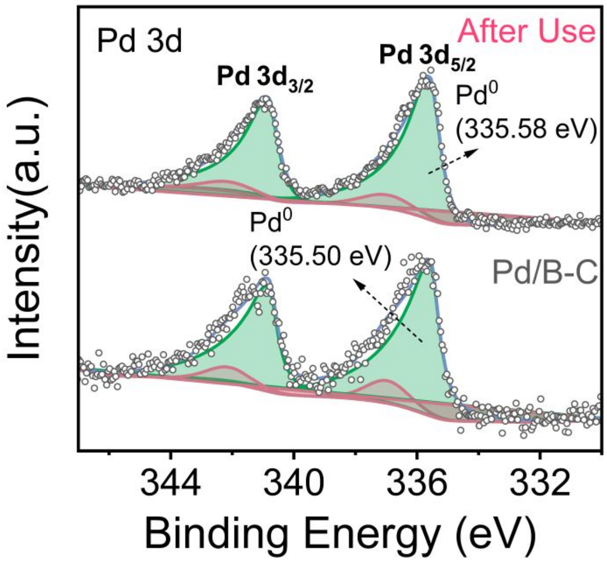

Fig. S33. The high-resolution XPS spectra of Pd 3d for freshly prepared Pd/B-C (Below) and Pd/B-C after FLO dehalogenation (Top).

After being used as cathode material for FLO dehalogenation, the ratio of Pd^0 in Pd/B-C slightly increases. This suggests that Pd^{2+} can be gradually reduced to Pd^0 at the potential (i.e., −1.0 V vs. SHE) for electrochemical FLO reduction, which is beneficial for retaining the cathodic dehalogenation activity. However, the binding energy for Pd^0 $3d_{5/2}$ slightly blue shifted after being used for FLO dehalogenation. This may be the reason for the slight performance drop after being used for 10 cycles.

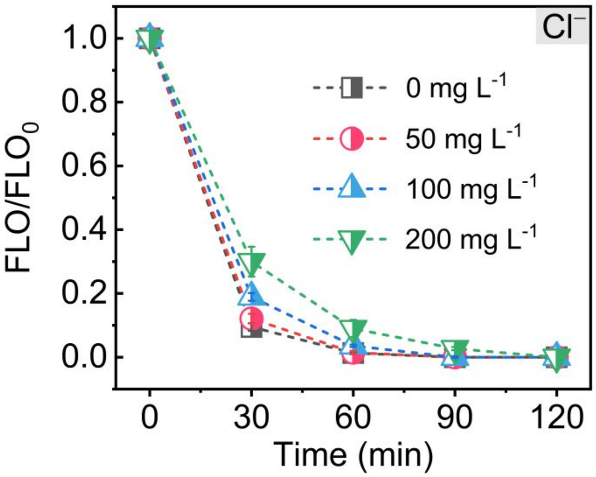

Fig. S34. FLO dehalogenation on Pd/B-C under different Cl^- concentrations. Conditions: 0.1 M Na_2SO_4 solution; C_0 = 20 mg L^{-1}; −1.0 V vs. SHE. Data are presented as mean ± s.d. (n = 3).

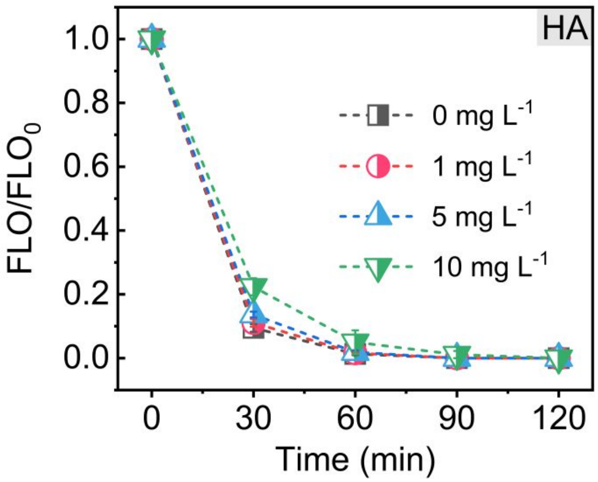

Fig. S35. FLO dehalogenation on Pd/B-C under different HA concentrations. Conditions: 0.1 M Na_2SO_4 solution; C_0 = 20 mg L^{-1}; −1.0 V vs. SHE. Data are presented as mean ± s.d. (n = 3).

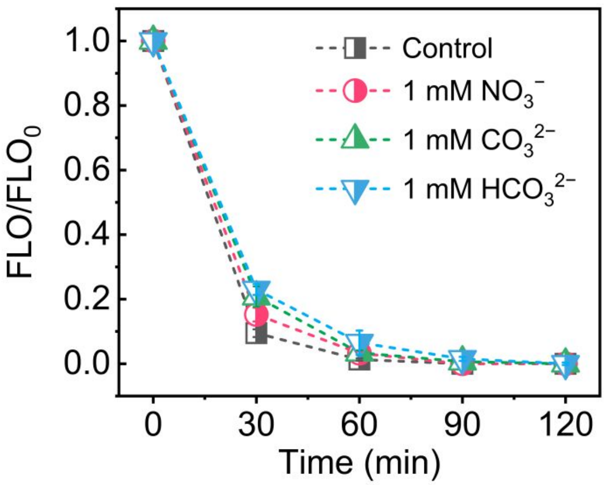

Fig. S36. FLO dehalogenation on Pd/B-C in the presence of different ions. Conditions: 0.1 M Na_2SO_4 solution; C_0 = 20 mg L^{-1}; −1.0 V vs. SHE. Data are presented as mean ± s.d. (n = 3).

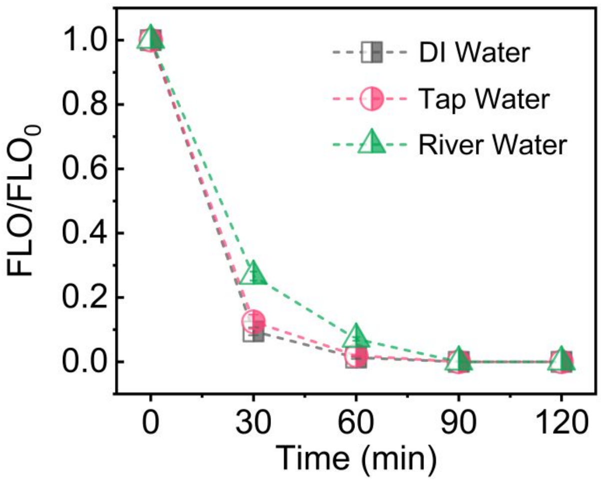

Fig. S37. FLO degradation in real water matrices. Conditions: C_0 = 20 mg L^{-1}; −1.0 V vs. SHE. Data are presented as mean ± s.d. (n = 3).

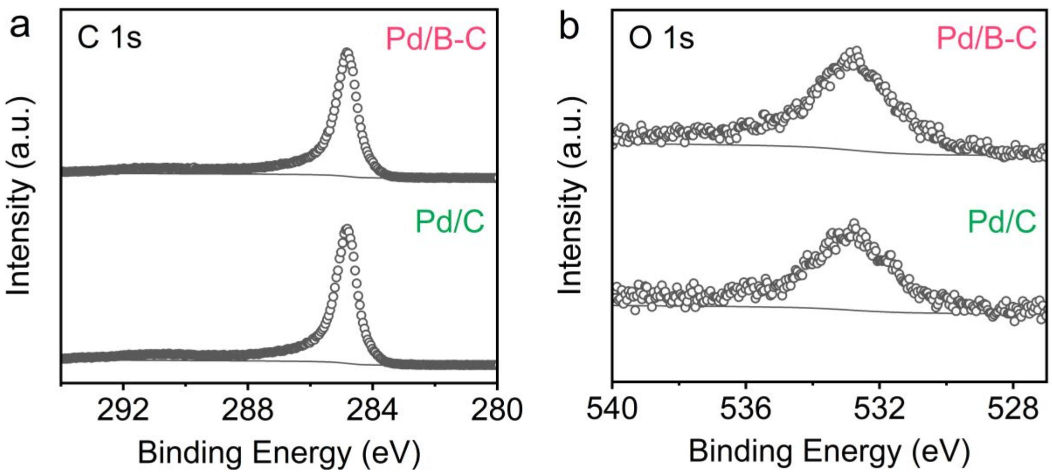

Fig. S38. The high-resolution XPS spectra of (a) C 1s and (b) O 1s for freshly prepared Pd/B-C and Pd/C.

The high resolution XPS spectra of C 1s and O 1s are characteristic of pristine graphene for both Pd/B-C and Pd/C [22].

Table S1. The relative Gibbs free energies of formation (G_f) and Boltzmann distribution for the global minimum and other top 15 conformers of FLO.

FLO (aq)	Relative G_f (kJ/mol)	Boltzmann distribution (%)
Global Minimum	0.00	53.63%
Conformer 1	7.56	2.54%
Conformer 2	9.88	1.00%
Conformer 3	7.96	2.17%
Conformer 4	1.70	27.07%
Conformer 5	9.02	1.41%
Conformer 6	4.47	8.85%
Conformer 7	22.76	0.01%
Conformer 8	22.98	0.01%
Conformer 9	7.67	2.44%
Conformer 10	11.05	0.62%
Conformer 11	15.98	0.09%
Conformer 12	14.30	0.17%
Conformer 13	22.71	0.01%
Conformer 14	20.96	0.01%

Table S2. FWHM of Pd(111) XRD diffraction peaks for different cathodes.

Material	FWHM (rad)
Pd/C	0.014
Pd/B(0.57)-C	0.020
Pd/B(0.71)-C	0.016
Pd/B(0.88)-C (i.e., Pd/B-C)	0.017

Since the FWHM is generally inversely proportional to the particle diameter. The results of FWHMs indicate that boron incorporation slightly reduces the particle sizes of cathodes.

Table S3. EXAFS data fitting results of Pd/C, Pd/B-C, and references.

Sample	Path	CN^a	R(Å)^b	σ^2 (Å^2)^c	ΔE_0(eV)^d	R factor
Pd K-edge (S_0^2=0.894)						
Pd foil	Pd-Pd	12*	2.740±0.002	0.0058	−5.4	0.0035
PdO	Pd-O	4.0±0.1	2.040±0.013	0.0026	1.7	0.0028
	Pd-O-Pd	4.1±0.3	3.056±0.018	0.0044		
	Pd-O-Pd	8.2±0.6	3.437±0.018	0.0060		
Pd/C	Pd-Pd	7.7±0.5	2.744±0.003	0.0059	−5.8	0.0066
Pd/B-C	Pd-C/B	1.3±0.2	1.954±0.005	0.0059	−6.0	0.0081
	Pd-Pd	6.7±0.4	2.749±0.011	0.0077		

^{a}*CN*, coordination number; ^{b}*R*, the distance between absorber and backscatter atoms; $^c\sigma^2$, the Debye Waller factor value; $^d\Delta E_0$, inner potential correction to account for the difference in the inner potential between the sample and the reference compound; *R* factor indicates the goodness of the fit. S_0^2 was fixed to 0.894, according to the experimental EXAFS fit of Pd foil by fixing *CN* as the known crystallographic value. *This value was fixed during EXAFS fitting, based on the known structure of Pd. Fitting conditions: *k* range: 3.0–14.0; *R* range: 1.0–3.0; fitting space: R space; *k*-weight = 3. A reasonable range of EXAFS fitting parameters: $0.800 < S_0^2 < 1.000$; $CN > 0$; $\sigma^2 > 0$ Å^2; $|\Delta E_0| < 15$ eV; R factor < 0.02.

Table S4. DFT calculated binding energies between Pd(111) and carbon or boron-doped carbon supports for different structures.

Structure	Binding Energy (eV)
Pd(111)/C	−0.72
Pd(111)/B-C	−1.95
Pd(111)/B2-C	−3.47

The DFT simulated binding energies between Pd(111) and carbon supports increase with B content.

Table S5. DFT calculated the change of Bader charge during the process of anchoring Pd on carbon or boron-doped carbon supports for different structures.

Structure	The Change of Bader Charge for Surface Pd Atoms (|e|)	The Change of Bader Charge for All Pd Atoms (|e|)	The Change of Bader Charge for B Atoms (|e|)
Pd(111)/C	0.005	−0.021	/
Pd(111)/B-C	0.13	−0.25	0.017
Pd(111)/B2-C	0.18	−0.28	0.0079

Compared to loading Pd(111) onto undoped carbon supports, loading Pd(111) onto boron-doped ones induces more electron transfer from bulk Pd atoms to carbon supports and the surface Pd atoms. The number of transferred electrons also increases with B content in carbon supports.

Table S6. Fitted R_{ct}[a] values by the ZView software.

	At Open Circuit Potential		At –1.0 V vs. SHE	
	With FLO	No FLO	With FLO	No FLO
GC	7188.0 Ω	9414.0 Ω	2620.0 Ω	1051.0 Ω
Pd/C	87.9 Ω	120.4 Ω	94.6 Ω	121.5 Ω
Pd/B-C	69.0 Ω	71.89 Ω	57.4 Ω	73.0 Ω

[a] R_{ct}: Charge-transfer resistance at the electrolyte electrode interface

Table S7. Comparison of catalytic activity of literature-reported cathodes on electrocatalytic FLO reduction.

Materials	Reaction conditions	Cathode area	Metal loading	FLO Removal	k_{obs} (min^{-1})	TOF[a] (min^{-1})	Mass activity[b] (mmol g^{-1} min^{-1})	Ref.
Pd/B-C	−1.0 V vs. SHE, 0.1 M Na_2SO_4, C_0: 20 mg L^{-1}, initial pH: 6, volume: 12 mL	1 cm^2	1 mg Pd/B-C cm^{-2}	60 min, 98.7%	0.072	0.57	1.11	This work
Commercial Pd/C	-1.0 V vs. SHE, 0.1 M Na_2SO_4, C_0: 20 mg L^{-1}, initial pH: 6, volume: 12 mL	1 cm^2	1 mg Pd/C cm^{-2}	120 min, 76.2%	0.012	/	0.19	
Pd/C-PEG25	−1.05V vs. Ag/AgCl, 50 mM Na_2SO_4, C_0: 179 mg L^{-1}, volume: 50 mL	2 cm^2	0.25 mg Pd cm^{-2}	180 min, ~ 82.0%	0.009	0.11	0.45	[40]

C-CoP/Ti	−1.2V vs. Ag/AgCl, 0.1 M Na_2SO_4, C_0: 20 mg L^{-1}, volume: 30 mL	0.96 cm^2	1.05 mg CoP cm^{-2}	30 min, 95.7%	0.1146	N.A.[d]	0.19	39
A-CoP/Ti	−1.2V vs. Ag/AgCl, 0.1 M Na_2SO_4, C_0: 20 mg L^{-1}, volume: 30 mL	0.96 cm^2	1.02 mg CoP cm^{-2}	30 min, 28.2%	0.0103	N.A.[d]	0.018	39
Ultrathin MoS_2	−1.2V vs. Ag/AgCl, 0.1 M Na_2SO_4, C_0: 20 mg L^{-1}, volume: 80 mL	3 cm^2	1.00 mg MoS_2 cm^{-2}	240 min, 96.0%	0.0135	N.A.[d]	0.02	41
Fe–CoP NWs/NiF	−1.2V vs. Ag/AgCl, 0.1 M Na_2SO_4, C_0: 20 mg L^{-1}, volume: 30 mL	1.8 cm^2	5.77 mg Fe-CoP cm^{-2}	30 min, 91.5%	0.0834	N.A.[d]	0.013	42

Fe–CoP NTs/NiF	−1.2V vs. Ag/AgCl, 0.1 M Na_2SO_4, C_0: 20 mg L^{-1}, volume: 30 mL	1.8 cm^2	4.49 mg Fe-CoP cm^{-2}	20 min, 100.0%	0.2748	N.A.[d]	0.057	[42]
MI-TiO_2-x	−1.2V vs. Ag/AgCl, 50 mM Na_2SO_4, C_0: 10 mg L^{-1}, initial pH: 5.7, volume: 100 mL	1.0 cm^2	0.94 mg TiO_2 cm^{-2}	180 min, 97.9%	0.021	/	0.062	[43]
TiO_2-x	−1.2V vs. Ag/AgCl, 50 mM Na_2SO_4, C_0: 10 mg L^{-1}, initial pH: 5.7, volume: 100 mL	1.0 cm^2	0.94 mg TiO_2 cm^{-2}	/	0.013	/	0.039	[43]
TiO_2	−1.2V vs. Ag/AgCl, 50 mM Na_2SO_4, C_0: 10 mg L^{-1}, initial pH: 5.7, volume: 100 mL	1.0 cm^2	0.94 mg TiO_2 cm^{-2}	/	0.009	/	0.027	[43]

MI-TiO_2	-1.2V vs. Ag/AgCl, 50 mM Na_2SO_4, C_0: 10 mg L^{-1}, initial pH: 5.7, volume: 100 mL	1.0 cm^2	0.94 mg TiO_2 cm^{-2}	/	0.011	/	0.033	43

[a] The turnover frequency (TOF, min^{-1}), the number of FLO molecules reduced per site per min, is calculated using Eq. S5 [22].

$$TOF = \frac{k_{obs} \cdot C_0}{C_M} \tag{S5}$$

Where, k_{obs} (min^{-1}) is the observed pseudo-first-order rate constant for FLO removal, C_0 (mol·L^{-1}) is the initial FLO concentration, and C_M (mol·L^{-1}) is the concentration of surface metal atoms. The ratio of the number of surface metal atoms to the number of total metal atoms for face-centered cubic (FCC) structure is calculated using Eq. S6 [44].

$$\frac{30n^2+6}{10n^3+15n^2+11n+3} \tag{S6}$$

Where n is the number of shells, $(2n+1)\cdot(atom\ diameter) = particle\ diameter$. The diameter of Pd atom was estimated to be 2.75 Å based on the reported lattice constant of Pd (3.89 Å) [45].

[b] Mass activity is calculated as the amount of disappeared FLO per unit of time and per unit of mass of the catalyst during the highest rate stage.

[c] The higher activity of our prepared Pd/C compared to other reported Pd-based cathodes can be attributed to the synergistic activity enhancement from: i) the hydrophilic surface [40], ii) the optimal annealing temperature (i.e., 900 ℃) of the carbon support [46], and iii) the promising methanol-mediated weakly-capping growth approach (WCGA) for growing Pd nanoparticles on the carbon support [47].

[d] Unable to calculate TOF due to lack of data on the number of available catalytic sites.

Table S8. Dehalogenation efficiencies for different cathode materials. Conditions: 0.1 M Na_2SO_4 solution; C_0 = 20 mg L^{-1}; −1.0 V vs. SHE.

Cathode Materials	Defluorination Ratio	Dechlorination Ratio
Pd/B-C	52.8%	98.0%
Pd/C	25.7%	91.4%
Commercial Pd/C	-	67.6%
Carbon Cloth	-	7.4%

Table S9. The summary of FLO defluorination ratios at different operating conditions. Common conditions: 0.1 M Na_2SO_4 solution; C_0 = 20 mg L^{-1}; −1.0 V vs. SHE. Only one of the factors (i.e., cathode potential, pH, *t*-BuOH concentration, and initial FLO concentrations) was varied at one time.

Cathode Potential (V vs. SHE)	Defluorination Ratio	pH	Defluorination Ratio[a]
−1.0	52.8%	2.2	26.3% (39.2%)
−0.9	46.2%	3.9	44.5% (49.3%)
−0.8	38.1%	5.8	51.5% (50.7%)
−0.6	20.1%	7.8	45.2% (48.6%)
−0.4	14.5%	9.5	29.7% (42.9%)
**Different *t*-BuOH Concentration (mg L^{-1})**	**Defluorination Ratio**	**Initial FLO Concentration (mg L^{-1})**	**Defluorination Ratio**
0	52.8%	1	N.A.
2	50.3%	10	86.5%
5	48.2%	20	52.8%
10	44.3%	50	69.3%

[a] the data in the parenthesis stands for those operated at −0.6 V vs. RHE.

Table S10. DFT calculated Pd-Pd length of surface Pd atoms for different structures.

Structure	Pd-Pd Length for Surface Pd atoms (Å)	Percentage of Strain (%)
Pd(111)	2.79	0.00
Pd(111)/C	2.81	0.73
Pd(111)/B-C	2.87	3.19
Pd(111)/B2-C	2.91	4.55

Boron doping in carbon supports induces stronger strain effects for surface Pd atoms as compared to undoped one. The extent of the strain effect also increases with B content in carbon supports.

Table S11. DFT calculated H* binding energies for different structures.

Structure	Binding Energy (eV)
Pd(111)/C	−0.67
Pd(111)B-C	−0.75
Pd(111)B2-C	−0.78

H* binding energies on Pd(111) surfaces increase with B content in carbon supports. This suggests stronger ability to preserve H* on cathodes with higher B content for higher cathodic dehalogenation performance.

Table S12. DFT calculated $\Delta G^{\ddagger}$ for HER for different structures.

Structure	$\Delta G^{\ddagger}$ (eV)
Pd(111)/C	1.05
Pd(111)/B-C	0.95
Pd(111)/B2-C	0.83

$\Delta G^{\ddagger}$ for HER, which generates H*, on Pd(111) surfaces decreases with B content in carbon supports. This suggests faster H* generation kinetics on cathodes with higher B content for higher cathodic dehalogenation performance.

Table S13. DFT calculated C-F cleavage E_a for different structures.

Structure	E_a for no H* (eV)	E_a for H* (eV)
Pd(111)/C	2.20	2.12
Pd(111)/B-C	1.96	1.85
Pd(111)/B2-C	1.86	1.79

E_a for C-F cleavage of FLO decreases with B content in carbon supports for both H* pre-adsorbed and no H* adsorbed Pd(111) surfaces, and H* pre-adsorbed surfaces have lower E_a than their no H* adsorbed counterparts. This suggests faster defluorination kinetics and higher defluorination performance on cathodes with higher B content and the enhancement effects of H* for defluorination. Notably, the E_a for no H* adsorbed Pd(111)/B-C was lower than that for H* pre-adsorbed Pd(111)/C. This indicates that B modification may endow Pd catalysts with H*-free direct C-F cleavage ability in addition to the primarily adopted H*-mediated hydrodefluorination mechanism.

Table S14. DFT calculated the number of transferred charges to FLO and F atom after FLO adsorption and Pd-F distance for different structures.

Structure	Charge Transfer to FLO (|e|)	Charge Transfer to F (|e|)	Pd-F distance (Å)
Pd(111)/C	0.10	0.0133	3.24
Pd(111)/B-C	0.13	0.0136	3.07
Pd(111)/B2-C	0.11	0.0138	3.01

Boron doping in carbon supports induces reduced Pd-F distances and more electron transfer from cathodes to F atoms in adsorbed FLO, which correlates with the higher defluorination efficiency for cathodes with higher B content.

Table S15. Water qualities for real water samples.

Water	Tap Water	River Water
pH	6.94	8.30
TOC (mg L^{-1})	0.45	9.00
Conductivity (mS cm^{-1})	0.182	0.796
Dissolved oxygen (mg L^{-1})	7.52	8.21
Ca^{2+} (mg L^{-1})	1.71	7.95
Na^{+} (mg L^{-1})	1.29	5.37
K^{+} (mg L^{-1})	1.15	1.31
Mg^{2+} (mg L^{-1})	0.41	1.80
Cl^{-} (mg L^{-1})	12.48	84.06
F^{-} (mg L^{-1})	0.16	0.31
NO_3^{-} (mg L^{-1})	3.78	8.59
SO_4^{2-} (mg L^{-1})	10.30	59.58
FLO degradation efficiency (60 min)	98.1 %	92.9 %

The river water has higher amounts of H* scavengers including TOC, Cl^{-}, and NO_3^{-} as compared to tap water. This correlates with the slightly lower FLO degradation efficiency in the river water.

Table S16. Comparison of energy consumption.

Process	Catalyst	FLO Concentration (mg L^{-1})	EE/O (kWh m^{-3})	Ref.
Electroreduction	Pd/B-C	20	0.20	This work
Electrooxidation	La-Ti_4O_7	1	1.91–29.3	[48]
Electrooxidation	Nb_2O_5/Ti	5	16.02	[49]
UV alone	N.A.	72	7236.18	[50]
UV/H_2O_2	N.A.	72	886.15	[50]
UV/PS	N.A.	72	415.58	[50]

[a] The electric energy per order (EE/O, kWh/m^3) represents the electric energy required for one log order of magnitude reduction of the target contaminant concentration in a unit volume and is given by Eq. S7.[51-53]

$$\mathbf{EE/O} = \frac{\boldsymbol{UI}\mathrm{t}}{\boldsymbol{V}\cdot\boldsymbol{log}\frac{\boldsymbol{C_0}}{\boldsymbol{C_t}}} \tag{S7}$$

Where, $\boldsymbol{U}$ and $\boldsymbol{I}$ are the applied voltage (volt) and current (A) of the electrochemical cell, respectively; $\boldsymbol{V}$ (L) is the volume of the electrochemical cell; $\boldsymbol{C_0}$ and $\boldsymbol{C_t}$ are the target contaminant concentrations at time = 0 h and time = t h, respectively.

SI References

1. Ravel, B.; Newville, M. ATHENA, ARTEMIS, HEPHAESTUS: data analysis for X-ray absorption spectroscopy using IFEFFIT. *J. Synchrotron Radiat.* **2005,** *12* (4), 537-541.
2. Zabinsky, S.; Rehr, J.; Ankudinov, A.; Albers, R.; Eller, M. Multiple-scattering calculations of X-ray-absorption spectra. *Phys. Rev. B* **1995,** *52* (4), 2995-3009.
3. Funke, H.; Chukalina, M.; Rossberg, A. Wavelet analysis of extended x-ray absorption fine structure data. *Phys. Scripta* **2005,** *71* (9), 232-234.
4. O'Boyle, N. M.; Vandermeersch, T.; Flynn, C. J.; Maguire, A. R.; Hutchison, G. R. Confab-Systematic generation of diverse low-energy conformers. *J. Cheminformatics* **2011,** *3* (1), 1-9.
5. Pracht, P.; Bohle, F.; Grimme, S. Automated exploration of the low-energy chemical space with fast quantum chemical methods. *Phys. Chem. Chem. Phys.* **2020,** *22* (14), 7169-7192.
6. Bannwarth, C.; Ehlert, S.; Grimme, S. GFN2-xTB—An Accurate and Broadly Parametrized Self-Consistent Tight-Binding Quantum Chemical Method with Multipole Electrostatics and Density-Dependent Dispersion Contributions. *J. Chem. Theory Computat.* **2019,** *15* (3), 1652-1671.
7. Grimme, S.; Bannwarth, C.; Shushkov, P. A robust and accurate tight-binding quantum chemical method for structures, vibrational frequencies, and noncovalent interactions of large molecular systems parametrized for all spd-block elements (Z = 1–86). *J. Chem. Theory Computat.* **2017,** *13* (5), 1989-2009.
8. Pracht, P., Caldeweyher, E., Ehlert, S. & Grimme, S. A robust non-self-consistent tight-binding quantum chemistry method for large molecules. *ChemRxiv* [Preprint]

(2019). https://doi.org/10.26434/chemrxiv.8326202.v1 (accessed 12 Dec 2023).

9. Lu, T. Molclus program, Version 1.9.9.9, http://www.keinsci.com/research/molclus.html (accessed on 12 Dec 2023)

10. Frisch, M. J.; Trucks, G. W.; Schlegel, H. B.; Scuseria, G. E.; Robb, M. A.; Cheeseman, J. R.; Scalmani, G.; Barone, V.; Petersson, G. A.; Nakatsuji, H.; Li, X.; Caricato, M.; Marenich, A. V.; Bloino, J.; Janesko, B. G.; Gomperts, R.; Mennucci, B.; Hratchian, H. P.; Ortiz, J. V.; Izmaylov, A. F.; Sonnenberg, J. L.; Williams; Ding, F.; Lipparini, F.; Egidi, F.; Goings, J.; Peng, B.; Petrone, A.; Henderson, T.; Ranasinghe, D.; Zakrzewski, V. G.; Gao, J.; Rega, N.; Zheng, G.; Liang, W.; Hada, M.; Ehara, M.; Toyota, K.; Fukuda, R.; Hasegawa, J.; Ishida, M.; Nakajima, T.; Honda, Y.; Kitao, O.; Nakai, H.; Vreven, T.; Throssell, K.; Montgomery Jr., J. A.; Peralta, J. E.; Ogliaro, F.; Bearpark, M. J.; Heyd, J. J.; Brothers, E. N.; Kudin, K. N.; Staroverov, V. N.; Keith, T. A.; Kobayashi, R.; Normand, J.; Raghavachari, K.; Rendell, A. P.; Burant, J. C.; Iyengar, S. S.; Tomasi, J.; Cossi, M.; Millam, J. M.; Klene, M.; Adamo, C.; Cammi, R.; Ochterski, J. W.; Martin, R. L.; Morokuma, K.; Farkas, O.; Foresman, J. B.; Fox, D. J. *Gaussian 16 Rev. B.01*, Wallingford, CT, 2016.

11. Zhao, Y.; Truhlar, D. G. The M06 suite of density functionals for main group thermochemistry, thermochemical kinetics, noncovalent interactions, excited states, and transition elements: two new functionals and systematic testing of four M06-class functionals and 12 other functionals. *Theor. Chem. Acc.* **2008,** *120* (1-3), 215-241.

12. Becke, A. D. Density-functional thermochemistry. I. The effect of the exchange-only gradient correction. *J. Chem. Phys.* **1992,** *96* (3), 2155–2160.

13. Kim, K.; Jordan, K. Comparison of density functional and MP2 calculations on the water monomer and dimer. *J. Phys. Chem.* **1994,** *98* (40), 10089-10094.

14. Stephens, P. J.; Devlin, F. J.; Chabalowski, C. F.; Frisch, M. J. Ab initio calculation of vibrational absorption and circular dichroism spectra using density functional force fields. *J. Phys. Chem.* **1994,** *98* (45), 11623-11627.

15. Weigend, F. Accurate Coulomb-fitting basis sets for H to Rn. *Phys. Chem. Chem. Phys.* **2006,** *8* (9), 1057-1065.

16. Weigend, F.; Ahlrichs, R. Balanced basis sets of split valence, triple zeta valence and quadruple zeta valence quality for H to Rn: Design and assessment of accuracy. *Phys. Chem. Chem. Phys.* **2005,** *7* (18), 3297-3305.

17. Clark, T.; Chandrasekhar, J.; Spitznagel, G. W.; Schleyer, P. V. Efficient diffuse function-augmented basis sets for anion calculations. III. the 3-21+G basis set for first-row elements. *J. Computat. Chem.* **1983,** *4* (3), 294-301.

18. Krishnan, R.; Binkley, J. S.; Seeger, R.; Pople, J. A. Self-consistent molecular-orbital methods. 20. basis set for correlated wave-functions. *J. Chem. Phys.* **1980,** *72* (1), 650-654.

19. Perdew, J. P.; Burke, K.; Ernzerhof, M. Generalized gradient approximation made simple. *Phys. rev. Lett.* **1996,** *77* (18), 3865.

20. Blöchl, P. E. Projector augmented-wave method. *Phys. Rev. B* **1994,** *50* (24), 17953.

21. Al-Gaashani, R.; Najjar, A.; Zakaria, Y.; Mansour, S.; Atieh, M. A. XPS and structural studies of high quality graphene oxide and reduced graphene oxide prepared by different chemical oxidation methods. *Ceram. Int.* **2019,** *45* (11), 14439-14448.

22. Huang, D.; Rigby, K.; Chen, W.; Wu, X.; Niu, J.; Stavitski, E.; Kim, J.-H. Enhancing the activity of Pd ensembles on graphene by manipulating coordination environment. *Proc. Natl. Acad. Sci.* **2023,** *120* (9), e2216879120.
23. Schmidt, T. O.; Ngoipala, A.; Arevalo, R. L.; Watzele, S. A.; Lipin, R.; Kluge, R. M.; Hou, S.; Haid, R. W.; Senyshyn, A.; Gubanova, E. L. Elucidation of structure–activity relations in proton electroreduction at Pd surfaces: Theoretical and experimental study. *Small* **2022,** *18* (30), 2202410.
24. Henkelman, G.; Uberuaga, B. P.; Jónsson, H. A climbing image nudged elastic band method for finding saddle points and minimum energy paths. *J. Chem. Phys.* **2000,** *113* (22), 9901-9904.
25. Wang, V.; Xu, N.; Liu, J.-C.; Tang, G.; Geng, W.-T. VASPKIT: A user-friendly interface facilitating high-throughput computing and analysis using VASP code. *Comput. Phys. Commun.* **2021,** *267*, 108033.
26. Tang, W.; Sanville, E.; Henkelman, G. A grid-based bader analysis algorithm without lattice bias. *J. Phys- Condens. Mat.* **2009,** *21* (8), 084204.
27. Lu, T.; Chen, Q. X., Shermo: A general code for calculating molecular thermochemistry properties. *Comput. Theor. Chem.* **2021,** *1200*.
28. Alecu, I. M.; Zheng, J. J.; Zhao, Y.; Truhlar, D. G. Computational thermochemistry: scale factor databases and scale factors for vibrational frequencies obtained from electronic model chemistries. *J. Chem. Theory Comput.* **2010,** *6* (9), 2872-2887.
29. Rappoport, D.; Furche, F. Property-optimized Gaussian basis sets for molecular response calculations. *J. Chem. Phys.* **2010,** *133* (13).

30. Pritchard, B. P.; Altarawy, D.; Didier, B.; Gibson, T. D.; Windus, T. L. New basis set exchange: An open, up-to-date resource for the molecular sciences community. *J. Chem. Inf. Model.* **2019,** *59* (11), 4814-4820.

31. Feller, D. The role of databases in support of computational chemistry calculations. *J. Comput. Chem.* **1996,** *17* (13), 1571-1586.

32. Schuchardt, K. L.; Didier, B. T.; Elsethagen, T.; Sun, L. S.; Gurumoorthi, V.; Chase, J.; Li, J.; Windus, T. L. Basis set exchange: A community database for computational sciences. *J. Chem. Inf. Model.* **2007,** *47* (3), 1045-1052.

33. Marenich, A. V.; Cramer, C. J.; Truhlar, D. G. Performance of SM6, SM8, and SMD on the SAMPL1 test set for the prediction of small-molecule solvation free energies. *J. Phys. Chem. B* **2009,** *113* (14), 4538-4543.

34. Zhang, Y. Y.; Moores, A.; Liu, J. X.; Ghoshal, S. New insights into the degradation mechanism of perfluorooctanoic acid by persulfate from density functional theory and experimental data. *Environ. Sci. Technol.* **2019,** *53* (15), 8672-8681.

35. Marenich, A. V.; Ho, J. M.; Coote, M. L.; Cramer, C. J.; Truhlar, D. G. Computational electrochemistry: prediction of liquid-phase reduction potentials. *Phys. Chem. Chem. Phys.* **2014,** *16* (29), 15068-15106.

36. Jing, Y.; Chaplin, B. P. Mechanistic study of the validity of using hydroxyl radical probes to characterize electrochemical advanced oxidation processes. *Environ. Sci. Technol.* **2017,** *51* (4), 2355-2365.

37. Kelly, C. P.; Cramer, C. J.; Truhlar, D. G. Aqueous solvation free energies of ions and ion-water clusters based on an accurate value for the absolute aqueous solvation free energy of the proton. *J. Phys. Chem. B* **2006,** *110* (32), 16066-16081.

38. Feng, J. B.; Ruan, H. T.; Chen, H. G.; Luo, J. Z.; Dong, J. D. Pharmacokinetics of florfenicol in the orange-spotted grouper, epinephelus coioides, following oral administration in warm seawater. *J. World Aquacult. Soc.* **2018,** *49* (6), 1058-1067.

39. Liu, H.; Han, J.; Yuan, J.; Liu, C.; Wang, D.; Liu, T.; Liu, M.; Luo, J.; Wang, A.; Crittenden, J. C. Deep dehalogenation of florfenicol using crystalline CoP nanosheet arrays on a Ti plate via direct cathodic reduction and atomic H. *Environ. Sci. Technol.* **2019,** *53* (20), 11932-11940.

40. Fan, Z.; Zhao, H.; Wang, K.; Ran, W.; Sun, J.-F.; Liu, J.; Liu, R. Enhancing electrocatalytic hydrodechlorination through interfacial microenvironment modulation. *Environ. Sci. Technol.* **2023,** *57* (3), 1499-1509.

41. Yang, J.; Jiang, S.-F.; Hu, W.-F.; Jiang, H. Highly efficient electrochemical dechlorination of florfenicol by an ultrathin molybdenum disulfide cathode. *Chem. Eng. J.* **2022,** *427*, 131600.

42. Liu, H.; Ding, Y.; Tang, H.; Du, Y.; Zhang, D.; Tang, Y.; Liu, C. Electrocatalytic deep dehalogenation of florfenicol using Fe-doped CoP nanotubes array for blocking resistance gene expression and microbial inhibition during biochemical treatment. *Water Res.* **2021,** *201*, 117361.

43. Lou, Z.; Wen, X.; Song, L.; Yan, C.; Chen, H.; Lu, T.; Yu, J.; Xu, X.; Li, J. Oxygen vacancy engineered molecular imprinted TiO_2 for preferential florfenicol remediation by electro-reductive approach: Enhanced dehalogenation performance and elimination of antibiotic resistance genes. *Appl. Catal. B Environ.* **2023,** *336*, 122923.

44. Klabunde, K. J.; Richards, R. M. *Nanoscale materials in chemistry*. John Wiley & Sons: 2009.

45. Jiang, B.; Zhang, X.-G.; Jiang, K.; Wu, D.-Y.; Cai, W.-B. Boosting formate production in electrocatalytic CO_2 reduction over wide potential window on Pd surfaces. *J. Am. Chem. Soc.* **2018,** *140* (8), 2880-2889.

46. Su, Y.; Yao, C.; Zhang, Q.; Xu, L.; Wang, H.; Liu, J.; Hou, S. Palladium nanoparticles supported on B-doped carbon nanocage as electrocatalyst toward ethanol oxidation reaction. *Chem. Electro. Chem.* **2019,** *6* (20), 5211-5219.

47. Zhu, Q.-L.; Tsumori, N.; Xu, Q. Immobilizing extremely catalytically active palladium nanoparticles to carbon nanospheres: a weakly-capping growth approach. *J. Am. Chem. Soc.* **2015,** *137* (36), 11743-11748.

48. Xu, J.; Liu, Y.; Li, D.; Li, L.; Zhang, Y.; Chen, S.; Wu, Q.; Wang, P.; Zhang, C.; Sun, J., Insights into the electrooxidation of florfenicol by a highly active La-doped Ti4O7 anode. *Sep. Purif. Technol.* **2022,** *291*, 120904.

49. Zhang, Y.-F.; Chen, S.-G.; Li, L.; Xu, J.-H.; Li, D.; Liu, Y.-F.; Quan, X.; Fu, X.; Xie, Y.-Z.; Wu, J.-N., Advanced electrooxidation of florfenicol using 3D printed Nb2O5/Ti electrodes: Degradation efficiency, dehalogenation performance, and toxicity reduction. *Chem. Eng. J.* **2023,** *474*, 145561.

50. Gao, Y.-Q.; Gao, N.-Y.; Deng, Y.; Yin, D.-Q.; Zhang, Y.-S., Degradation of florfenicol in water by UV/Na 2 S 2 O 8 process. *Environ. Sci. Pollut. R.* **2015,** *22*, 8693-8701.

51. Kokate, S.; Gupta, S.; Kopuri, V. G.; Prakash, H., Energy efficient photocatalytic activation of peroxymonosulfate by g-C3N4 under 400 nm LED irradiation for degradation of Acid Orange 7. *Chemosphere* **2022,** *287*, 132099.

52. Lee, C.-S.; Venkatesan, A. K.; Walker, H. W.; Gobler, C. J., Impact of groundwater quality and associated byproduct formation during UV/hydrogen peroxide treatment of 1,4-dioxane. *Water Res.* **2020,** *173*, 115534.

53. Lou, Z.; Wang, J.; Wang, S.; Xu, Y.; Wang, J.; Liu, B.; Yu, C.; Yu, J., Strong hydrophobic affinity and enhanced •OH generation boost energy-efficient electrochemical destruction of perfluorooctanoic acid on robust ceramic/PbO2-PTFE anode. *Sep. Purif. Technol.* **2022,** *280*, 119919.
